# Dnmt3a Regulates Proliferation of Muscle Satellite Cells via p57Kip2

**DOI:** 10.1371/journal.pgen.1006167

**Published:** 2016-07-14

**Authors:** Masashi Naito, Masaki Mori, Masayo Inagawa, Kohei Miyata, Naohiro Hashimoto, Sakae Tanaka, Hiroshi Asahara

**Affiliations:** 1 Department of Systems BioMedicine, Tokyo Medical and Dental University, Bunkyo-Ku, Tokyo, Japan; 2 Department of Orthopaedic Surgery, The University of Tokyo, Bunkyo-ku, Tokyo, Japan; 3 Department of Systems BioMedicine, National Center for Child Health and Development, Setagaya-Ku, Tokyo, Japan; 4 Department of Regenerative Medicine, National Institute for Longevity Sciences, National Center for Geriatrics and Gerontology, Morioka, Oobu, Aichi, Japan; 5 Japan Agency for Medical Research and Development, the Core Research for the Evolutionary Science and Technology, Chiyoda-ku, Tokyo, Japan; 6 Department of Molecular and Experimental Medicine, The Scripps Research Institute, La Jolla, California, United States of America; Pasteur Institute, FRANCE

## Abstract

Cell differentiation status is defined by the gene expression profile, which is coordinately controlled by epigenetic mechanisms. Cell type-specific DNA methylation patterns are established by chromatin modifiers including *de novo* DNA methyltransferases, such as *Dnmt3a* and *Dnmt3b*. Since the discovery of the myogenic master gene *MyoD*, myogenic differentiation has been utilized as a model system to study tissue differentiation. Although knowledge about myogenic gene networks is accumulating, there is only a limited understanding of how DNA methylation controls the myogenic gene program. With an aim to elucidate the role of DNA methylation in muscle development and regeneration, we investigate the consequences of mutating *Dnmt3a* in muscle precursor cells in mice. *Pax3* promoter-driven *Dnmt3a*-conditional knockout (cKO) mice exhibit decreased organ mass in the skeletal muscles, and attenuated regeneration after cardiotoxin-induced muscle injury. In addition, *Dnmt3a*-null satellite cells (SCs) exhibit a striking loss of proliferation in culture. Transcriptome analysis reveals dysregulated expression of *p57Kip2*, a member of the Cip/Kip family of cyclin-dependent kinase inhibitors (CDKIs), in the *Dnmt3a*-KO SCs. Moreover, RNAi-mediated depletion of *p57Kip2* replenishes the proliferation activity of the SCs, thus establishing a role for the *Dnmt3a*-*p57Kip2* axis in the regulation of SC proliferation. Consistent with these findings, *Dnmt3a*-cKO muscles exhibit fewer Pax7^+^ SCs, which show increased expression of p57Kip2 protein. Thus, Dnmt3a is found to maintain muscle homeostasis by epigenetically regulating the proliferation of SCs through *p57Kip2*.

## Author Summary

How muscle homeostasis is maintained is not completely elucidated yet. Epigenetic disorders such as Beckwith-Wiedemann syndrome, which causes hypergrowth of skeletal muscles and rhabdomyosarcoma, indicate that epigenetic regulations such as DNA methylation, contribute to this homeostasis control. DNA methylation is mediated by DNA methyltransferases, such as Dnmt3a and Dnmt3b, which are de novo DNA methyltransferases. The role of DNA methylation in somatic stem cells is not completely understood, although it has been shown to be indispensable in differentiation of primordial germ cells and embryonic stem cells. In this report, we investigated the role of Dnmt3a in muscle satellite cells by analyzing *Dnmt3a*-conditional knockout (cKO) mice in which *Dnmt3a* loci are deleted utilizing Cre-recombinase driven by *Pax7* or *Pax3* promoters that are specifically activated in the muscle precursor lineage. The loss of Dnmt3a in cKO mice causes decreased muscle mass and significantly impaired muscle regeneration. Moreover, Dnmt3a loss also results in a striking loss of proliferation of SCs, which is caused by mis-expression of a cyclin-dependent kinase inhibitor, *p57Kip2*. Therefore, our findings suggest that DNA methylation plays an essential role in muscle homeostasis.

## Introduction

Myogenic differentiation program has been extensively studied as a model of tissue differentiation since the discovery of *MyoD* [1]. Although much is known about the gene cascade of myogenesis [2,3], the epigenetic mechanisms that regulate the physiological and pathological condition of skeletal muscles remain unknown [4].

Gene expression is regulated by both genetic and epigenetic mechanisms. DNA methylation is an epigenetic modification, which usually occurs at CpG sites [5]; the cytosine residues at CpG sites are methylated to 5-methyl-cytosine. This DNA methylation is mediated by a group of DNA methyltransferases (Dnmt) [6]. Among them, Dnmt3a and Dnmt3b catalyze *de novo* DNA methylation, and Dnmt1 mediates the maintenance of DNA methylation [7–9]. Accumulating evidence suggests that DNA methylation by Dnmt proteins in the promoter regions is associated with gene silencing, thus linking DNA methylation to gene suppression [6,10]. Recent studies have also clarified the roles of DNA methylation in gene bodies and intergenic regions in enhancing gene expression [11–14].

We previously reported that a transcriptional repressor Rp58, which has been known to bind Dnmt3a [15], is a direct target of MyoD and has an essential role in skeletal myogenesis [16], in which DNA methylation at the promoter of myogenic genes is implicated [17].

*Dnmt3a*-null mice die within 3 to 4 weeks after birth, and deletion of *Dnmt1* or *Dnmt3b* leads to early embryonic lethality [9,18,19], indicating that DNA methylation has a critical role in embryogenesis and postnatal homeostasis. The Dnmt1-mediated maintenance of DNA methylation is necessary for self-renewal of the hematopoietic, mammary, mesenchymal and skin stem cells [20–23]. On the other hand, Dnmt3a and Dnmt3b coordinately generate DNA methylation profiles in differentiating stem cells, resulting in determination of distinct cell fates. In embryonic stem cells, concomitant deletion of *Dnmt3a* and *Dnmt3b* leads to a loss of differentiation capacity [24].

The precise role of *de novo* DNA methylation by Dnmt3a and Dnmt3b in muscle SCs, however, remains to be characterized. Hematopoietic stem cells null for *Dnmt3a* and/or *Dnmt3b*, progressively lose differentiation potential [25,26] and self-renewal capacity [27]. Neural stem cells deficient for *Dnmt3a* show impaired differentiation and increased cell proliferation [28], and *Nestin-*Cre-mediated deletion of *Dnmt3a* causes motor neuron defects and premature death of the mice [29]. *Dnmt3a*-deficient osteoclast precursor cells do not differentiate into osteoclasts efficiently [30]. However, little is known about the functions of Dnmt3a in the muscle SCs.

Proper muscle development and regeneration require coordinated gene expressions in embryonic muscle precursor cells and adult SCs [2,4]. The embryonic muscle precursor cells originate from dermomyotome, a dorsal part of the somite, which gives rise to myotome and dermatome. During embryogenesis, muscle precursor cells expressing Paired box 3 (Pax3) transcription factor appear in dermomyotome. These Pax3^+^ cells are myogenic progenitor cells and a portion of them also express Pax7. Most of the Pax3^+^/Pax7^+^ cells, and Pax3^+^/Pax7^-^ cells are defined as myoblasts in later stages and develop into skeletal muscles. A small fraction of the Pax3^+^/Pax7^+^ cells becomes quiescent and settle in as SCs in postnatal skeletal muscles [31–33]. The myoblasts express muscle regulatory factors (MRFs) such as *Myf5*, *MyoD*, *Myogenin* (*Myog)* and *Mrf4*, and then differentiate and fuse with each other to form myotubes, which mature into myofibers [34]. *Pax3*-null mice are devoid of all limb muscles [35].

In the muscle tissues, SCs are located on the surface of myofibers, inside the ensheathing basal lamina, and regulated by both extrinsic and intrinsic factors [36–38]. In the steady state, SCs maintain quiescence and express *Pax7* [31]. Upon muscle injury, they are activated and proliferate to form muscle fibers for regeneration [39]. Upon activation, *Pax7* expression is rapidly lost and the MRFs are induced during regeneration. SCs are also responsible for postnatal muscle growth [40], and age-related muscle decline is associated with functional impairment of SCs [38].

The number of tissue precursor cells increases during organ development and tissue regeneration. The precise mechanism underlying the proliferation of SCs is not fully understood. Cell cycle is regulated by a set of cell cycle factors, including Cyclins, Cyclin-dependent kinases (CDKs), and CDK inhibitors (CDKIs). CDKIs, the negative regulators of cell cycle, comprise two families, namely the INK4 and the Cip/Kip families. Members of the INK4 family (p16INK4a, p15INK4b, p18INK4c and p19INK4d) inhibit CDK4 and CDK6, whereas Cip/Kip members (p21Cip1, p27Kip1, and p57Kip2) mainly inhibit CDK2 and CDK4 [41]. Among them, p57Kip2 (also called as Cdkn1c) is reportedly important to maintain the hematopoietic stem cells in a non-proliferative state [42,43]. The *p57Kip2* is located at an imprinted locus and loss-of-function mutations in *p57Kip2* cause Beckwith-Wiedemann syndrome, an overgrowth disorder which is characterized by increased organ sizes including that of muscles [44,45], and gain-of-function mutations cause undergrowth disorders such as Silver-Russell syndrome [46–48].

Here, we show an indispensable role of Dnmt3a in muscle SCs by utilizing muscle precursor cell-specific *Dnmt3a* deletion in mice, and identify *p57Kip2* as a critical target gene of Dnmt3a for the proper proliferation of SCs.

## Results

### Loss of *Dnmt3a* causes decreased muscle mass in mice

**Fig 1:**
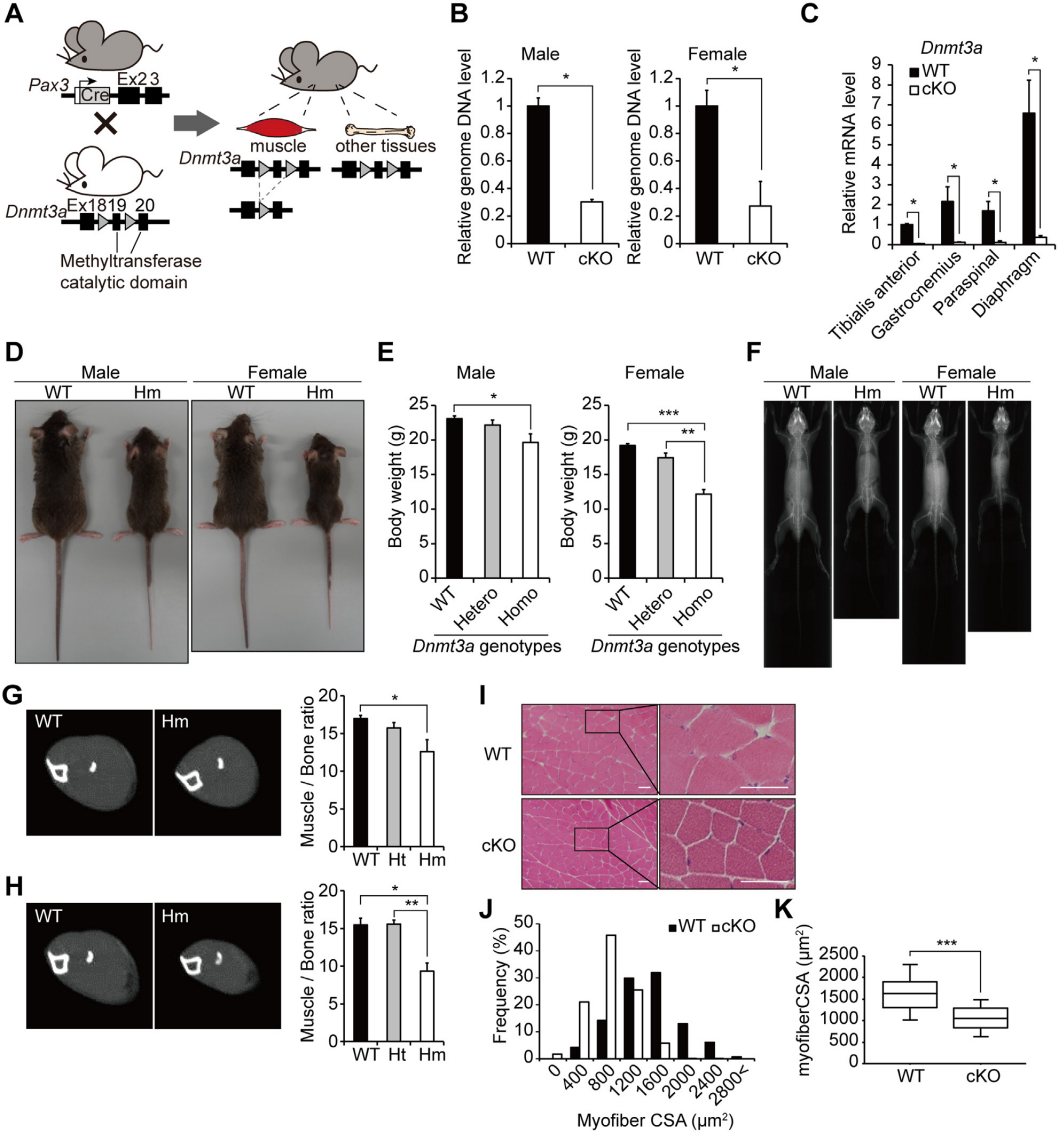
Loss of *Dnmt3a* causes reduced muscle mass in mice. (A) A schematic diagram of muscle precursor cell-specific *Dnmt3a* deletion by Cre-loxP recombination. Triangles represent loxP sites. (B) Genomic DNA levels of *Dnmt3a* in tibialis anterior muscles; *Col2a1* promoter served as the control; *p<0.05, Student’s t-test. (C) RT-qPCR analysis for *Dnmt3a* using muscle tissues; *Gapdh* served as the control; *p<0.05, Student’s t-test. (D) *Dnmt3a*-cKO mice have smaller body sizes than WT controls. (E) *Dnmt3a*-cKO mice weigh less than WT controls; *p<0.05, **p<0.01, ***p<0.001, Student’s t-test. (F) X-ray whole body imaging of *Dnmt3a*-cKO and WT mice; (G, H) Muscle mass in male (G) and female (H) mice. (Left) CT images of the lower hindlimb of *Dnmt3a*-cKO and WT mice. CT images of the slices that have the maximum muscle CSA in each limb are shown. (Right) The ratio of maximum CSA of the muscle divided by that of the bone at the same slice level demonstrates disproportionately reduced muscle mass in *Dnmt3a*-cKO mice; *p<0.05, **p<0.01, Student’s t-test. (I) HE staining of the gastrocnemius muscle cross sections of *Dnmt3a*-cKO and WT mice. (J) Distribution of myofiber CSAs in *Dnmt3a*-cKO and WT muscles. (K) Box plots for myofiber CSAs in *Dnmt3a*-cKO and WT muscles; ***p<0.001, Mann-Whitney U test. Data represent mean ± SEM. Hm—*Dnmt3a* homozygous knockout; Ht—*Dnmt3a* heterozygous knockout. Scale bar—200 μm.

To assess the role of DNA methylation in muscle development, we analyzed muscle precursor cell-specific *Dnmt3a* cKO mice. We established a mouse line in which *Dnmt3a* gene was deleted by Cre recombinase driven by a *Pax3* promoter (Fig 1A). The efficiency of deletion in tibialis anterior muscles of cKO mice was approximately 70% at the genomic DNA level (Fig 1B), and over 90% at the mRNA level in tibialis anterior, gastrocnemius, paraspinal muscles and diaphragm (Fig 1C); *Dnmt3b* expression level was unaffected (S1A Fig). The *Dnmt3a*-cKO mice exhibited significantly smaller body sizes than WT littermates at 8- to 12-week old (Fig 1D), although they were born at normal Mendelian ratios, and were viable. The *Dnmt3a*-cKO mice weighed less than WT controls and the difference was more prominent in females (Fig 1E). No apparent skeletal deformity was observed using X-ray whole body imaging (Fig 1F). Muscle tissues were hypoplastic in *Dnmt3a*-cKO mice (S1B Fig). Computed Tomography (CT) scan of distal hindlimbs revealed significantly reduced muscle mass in the *Dnmt3a*-cKO mice compared to WT controls (Fig 1G and 1H), and the difference was more prominent in females (Fig 1G and 1H). Histological analysis of the gastrocnemius muscles revealed that myofibers in *Dnmt3a*-cKO muscles were narrower than WT myofibers (Fig 1I and 1J). Median myofiber cross sectional area (CSA) of the *Dnmt3a*-cKO muscles was significantly smaller than that of the WT muscles (Fig 1K). Growth retardation and decreased muscle mass in *Dnmt3a*-cKO mice persisted at later stages as well and growth did not catch up with WT littermates. These findings indicate that the loss of *Dnmt3a* in muscles leads to reduced muscle mass. The relatively well- maintained muscle tissue patterns prompted us to investigate the status of muscle differentiation. Gene expression analysis in muscles did not reveal any significant differences in myogenic gene expression between *Dnmt3a*-cKO and WT muscles (S1C Fig), suggesting that *Dnmt3a* deletion does not affect myogenic differentiation. These findings suggest that the loss of *Dnmt3a* in the Pax3^+^ myogenic precursor cells leads to decreased muscle mass in mice.

### *Dnmt3a*-cKO mice show impaired muscle regenerative capacity

**Fig 2:**
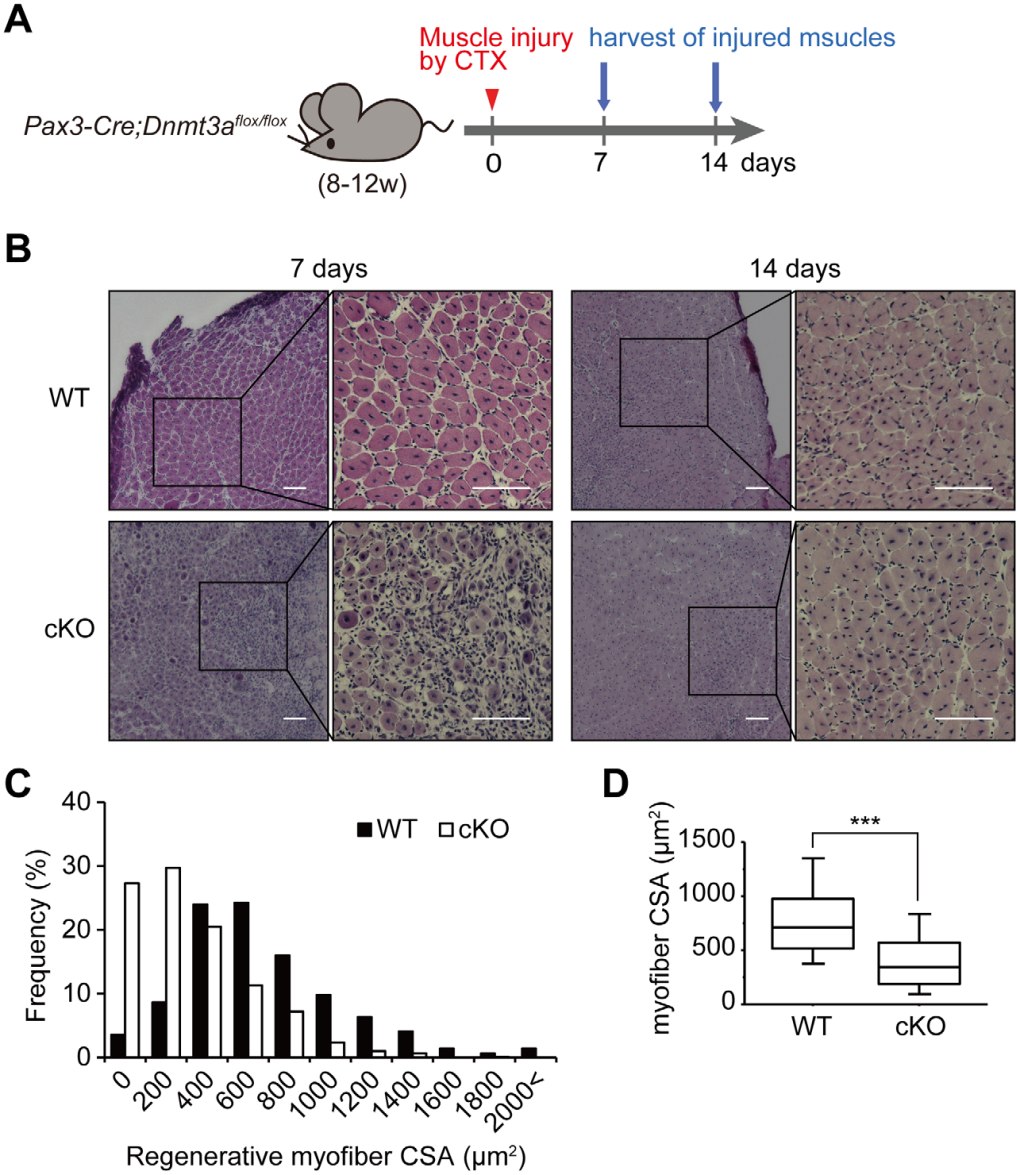
*Dnmt3a*-cKO mice show impaired muscle regenerative capacity. (A) A schematic diagram of the muscle injury and regeneration experiment. (B) HE-stained sections of the tibialis anterior muscle of *Dnmt3a*-cKO and WT mice, 7 and 14 days after cardiotoxin (CTX) injection. Centrally nucleated regenerative myofibers are scarce and thin in the *Dnmt3a*-cKO muscle 7 days after injury, compared to WT. (C) Distribution of regenerative myofiber CSAs 7 days after injury. (D) Box plots for regenerative myofiber CSAs 7 days after injury indicate significantly smaller myofiber CSA in *Dnmt3a*-cKO muscle than WT; ***p<0.001, Mann-Whitney U test. Scale bar, 100 μm.

The finding that *Dnmt3a*-cKO muscles are hypoplastic implied that the potential of muscle precursor cells to grow organs had reduced. To investigate the role of muscle SCs in recreating muscle tissues, we probed muscle regeneration in the cKO mice (Fig 2A). The tibialis anterior muscles were injected with cardiotoxin (CTX) to induce tissue injury. Histological analysis of the muscles 7 days after the CTX treatment revealed smaller regenerated myofibers with a central nucleus, in the *Dnmt3a*-cKO muscles than in the WT muscles (Fig 2B and 2C). Median regenerative myofiber CSA of *Dnmt3a*-cKO muscles was significantly smaller than that of WT muscles (Fig 2D). These findings indicate that muscle regenerative capacity is impaired in *Dnmt3a*-cKO mice. Since the loss of *Dnmt3a* causes decreased muscle formation in adult mice also, it implies that *Dnmt3a* loss impairs the function of adult SCs.

### Loss of *Dnmt3a* leads to impaired proliferation of muscle satellite cells

To gain a mechanistic insight into how loss of *Dnmt3a* leads to a functional impairment of the SCs, we performed an *in vitro* analysis of the muscle SCs. We isolated SCs from *Pax3-Cre; Dnmt3a*-cKO mice and WT littermates and cultured the cells in growth conditions. The proliferation of *Dnmt3a*-cKO SCs was impaired relative to that of WT SCs, indicating that Dnmt3a is required for SCs to re-enter the cell cycle (S2A and S2B Fig). Because Pax3 is expressed during development, we considered that there may be an effect of *Pax3*-dependent *Dnmt3a* deletion during the development of SCs.

**Fig 3:**
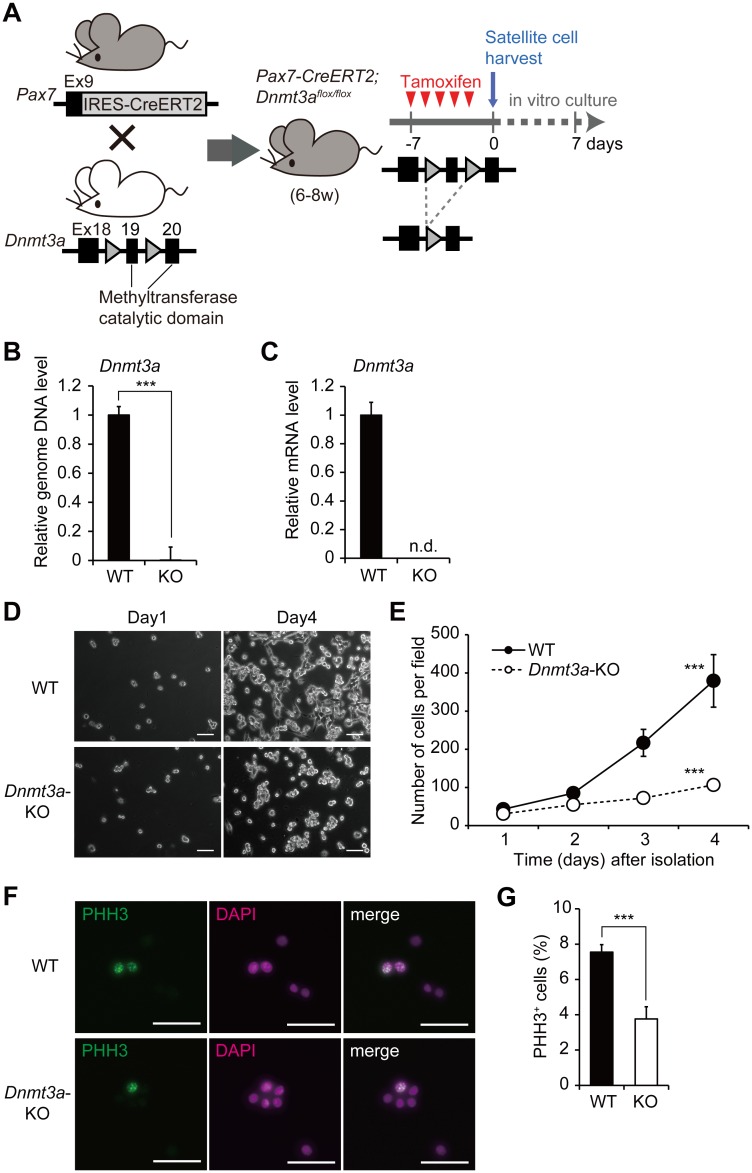
Loss of *Dnmt3a* leads to impaired proliferation of muscle satellite cells. (A) A schematic diagram of conditional *Dnmt3a*-KO in muscle SCs. Triangles represent loxP sites. (B) Genomic *Dnmt3a* levels in the SCs; *Col2a1* promoter served as the control; ***p<0.001, Student’s t-test. (C) *Dnmt3a* mRNA levels in the SCs; *Gapdh* served as the control. *Dnmt3a* expression in cKO SCs is below detectable levels. (D) Representative phase-contrast microscopic images of *Dnmt3a*-KO and WT SCs. Both KO and WT cells were disseminated at the same cell density on Day 0. Scale bar—200 μm. (E) *In vitro* cell proliferation assay shows significantly reduced proliferation of *Dnmt3a*-KO SCs compared to WT SCs; ***p<0.001, two-way repeated measures ANOVA. (F) Representative images of PHH3 immunocytochemistry of *Dnmt3a*-KO and WT SCs. Arrowheads indicate PHH3^+^ cells. Scale bar—30 μm. (G) Quantification of PHH3^+^ cells in *Dnmt3a*-KO and WT SCs; ***p<0.001, Student’s t-test. Data represent mean ± SEM.

In our evaluation of the non-muscle effects of the *Pax3* promoter-dependent *Dnmt3a* mutation, we found that *Pax7*-KO mice, which completely lack SCs, exhibit growth retardation and thin myofibers, indicating that dysfunction in SCs leads to growth retardation [40]. Accordingly, we considered that the *Dnmt3a*-cKO mouse phenotype was attributable to impaired SC function. To eliminate the possible developmental deficit of SCs and non-muscle effects, we utilized a tamoxifen-inducible *Pax7-CreERT2* system and generated *Pax7-CreERT2; Dnmt3a*^*flox/flox*^ mice for later analyses. *Pax7-Cre; Dnmt3a*-KO SCs were isolated from *Pax7-CreERT2; Dnmt3a*^*flox/flox*^ mice after tamoxifen injection (Fig 3A). *Dnmt3a* KO efficiency was over 99% both at the genomic DNA level (Fig 3B) and mRNA level (Fig 3C). The morphologies of the isolated *Dnmt3a*-KO SCs were indistinguishable from those of WT SCs (Fig 3D, Day 1). However, *Dnmt3a*-KO SCs showed a striking loss of expansion in culture and their growth rate was significantly lower than that of WT SCs (Fig 3D and 3E). To explore whether the impaired expansion of *Dnmt3a*-KO SCs was caused by decreased proliferation of the SCs, we performed phosphorylated histone H3 (PHH3-Ser10) immunostaining of the SCs. The frequency of the PHH3-Ser10^+^ *Dnmt3a*-KO SCs was significantly lower than that of WT SCs (Fig 3F and 3G). We also performed 5-ethynyl-20-deoxyuridine (EdU) incorporation assay. EdU^+^ cells were significantly less frequent in *Dnmt3a*-KO SCs than in WT SCs (S4 Fig). These findings suggest that cell proliferation is impaired in *Dnmt3a*-KO SCs. With regard to apoptosis, we immunostained proliferating *Pax7-Cre; Dnmt3a*-cKO and WT SCs with a cleaved Caspase-3 antibody. The frequency of cleaved Caspase-3-positivity was very low in *Dnmt3a*-cKO SCs and not statistically different from that in WT SCs. These results suggest that the loss of expansion observed in *Dnmt3a*-KO SCs was attributable not to activated apoptosis but to decreased proliferation (S5 Fig). To examine the influence of the *Dnmt3a* deletion on the differentiation capacity of SCs, myogenic differentiation was induced by serum starvation. The number of cells was strictly adjusted so that differentiation was induced at the same confluency in both *Dnmt3a*-KO and WT SCs. The *Dnmt3a*-KO SCs showed no apparent morphological differences from WT SCs (S6A Fig). Also, the expression of myogenic genes was not different significantly, indicative of the unaffected myogenic differentiation capacity of the *Dnmt3a*-KO SCs, compared to the WT SCs (S6B Fig). Collectively, loss of *Dnmt3a* leads to decreased proliferation of muscle SCs.

### Loss of *Dnmt3a* leads to elevated expression of *p57Kip2* in SCs

**Fig 4:**
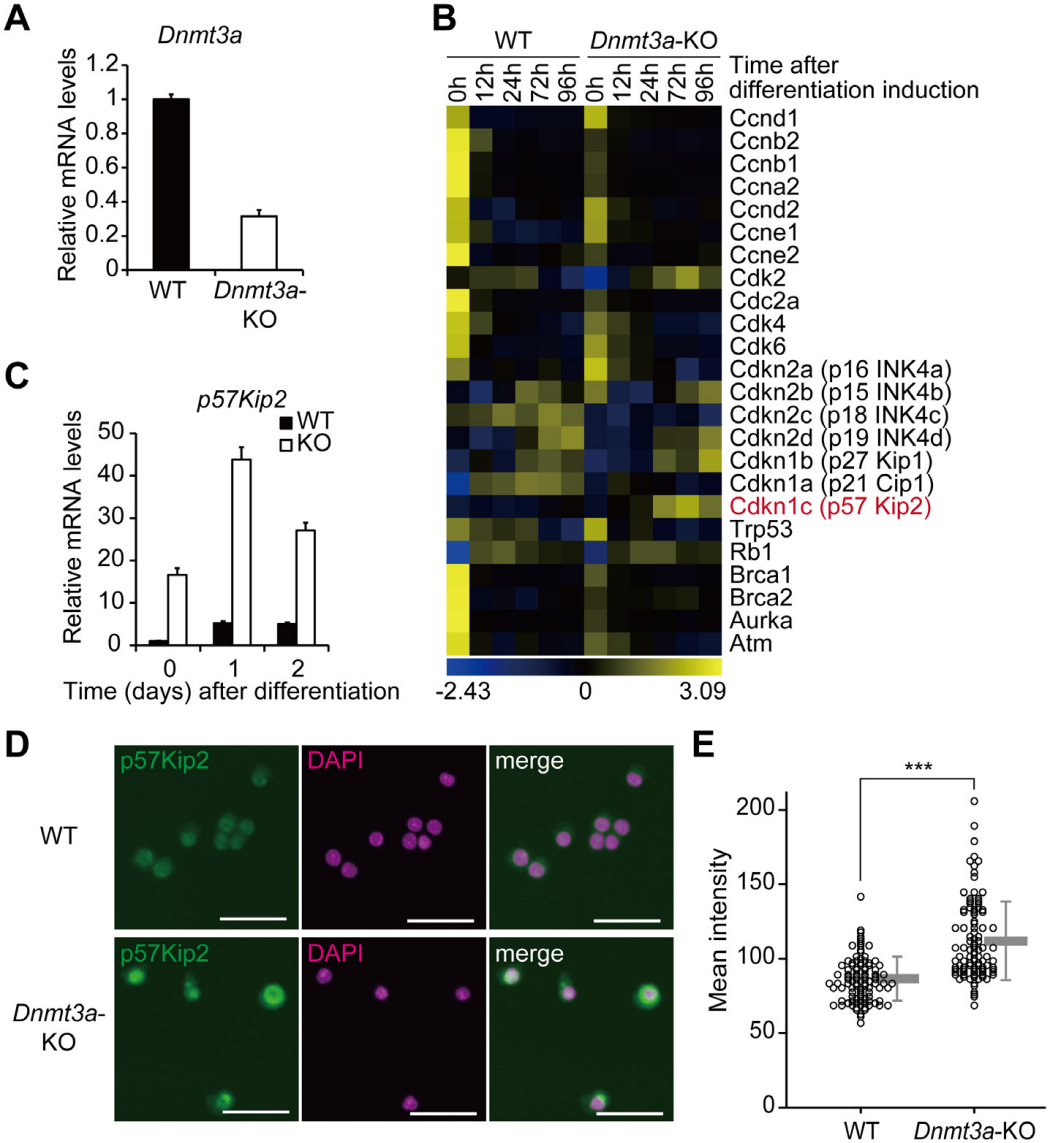
Loss of *Dnmt3a* leads to elevated expression of *p57Kip2*. (A) RT-qPCR analysis of *Dnmt3a* in Ax-Cre *Dnmt3a*-KO and WT SCs before differentiation. (B) A heat map showing the expression levels of cell cycle-regulating genes based on the transcriptome analysis of Ax-Cre *Dnmt3a*-KO and WT SCs. *p57Kip2* (*Cdkn1c*) is highly expressed in *Dnmt3a*-KO SCs. (C) RT-qPCR analysis of *p57Kip2* in *Pax7-Cre; Dnmt3a*-KO and WT SCs. (D) Representative photomicrographs of *Pax7-Cre; Dnmt3a*-KO and WT SCs stained with p57Kip2 and DAPI. Scale bar—30 μm. (E) Dot plots of mean signal intensities of fluorescence in each SC stained with an anti-p57Kip2 antibody; Gray bars represent mean ± SD, ***p<0.001, Student’s t-test.

To elucidate the mechanism of how Dnmt3a regulates the proliferative capacity of SCs, we performed transcriptome analysis of *Dnmt3a*-KO SCs. To minimize the potential developmental differences in the SCs of the *Dnmt3a*-cKO mice, we established a temporal deletion of *Dnmt3a* by infecting *Dnmt3a*^*flox/flox*^ SCs with adenovirus expressing Cre-recombinase (Ax-Cre). The *Dnmt3a* deletion efficiency was approximately 70% at the mRNA level (Fig 4A). Consistent with the gene expression analysis in the *Pax7*-dependent deletion of *Dnmt3a*, the expression of myogenic genes was not significantly altered in the Ax-Cre-mediated *Dnmt3a*-KO SCs (S7A Fig). Among cell-cycle related genes, the expression of *p57Kip2*, a negative regulator of cell cycle, increased in the Ax-Cre *Dnmt3a* KO SCs without induction of differentiation (Fig 4B). The increased expression of *p57Kip2* was also observed in the *Pax7*-dependent *Dnmt3*a-KO SCs (Fig 4C), and it continued even after differentiation (Fig 4C). Immunostaining with a p57Kip2 antibody showed significantly higher intensities of fluorescence in *Pax7-Cre; Dnmt3a*-cKO SCs than in WT SCs, suggesting enhanced expression of p57Kip2 in the *Pax7-Cre; Dnmt3a*-cKO SCs (Fig 4D and 4E). According to RT-qPCR analysis of *Pax7-Cre*; *Dnmt3*a-KO and WT SCs for all of the other CDKIs, the expression level of *p16INK4a* was only elevated by *Dnmt3a* loss (S8 Fig). But the difference of *p16INK4a* expression between *Dnmt3a*-KO and WT SCs was much smaller than that of *p57Kip2*. Therefore, we considered p57Kip2 as a primary candidate of a causative factor of impaired proliferation of *Dnmt3a*-KO SCs. Collectively, loss of *Dnmt3a* leads to elevated expression of *p57Kip2* in SCs.

### *p57Kip2* is a methylation target of Dnmt3a and regulates proliferation of SCs

**Fig 5:**
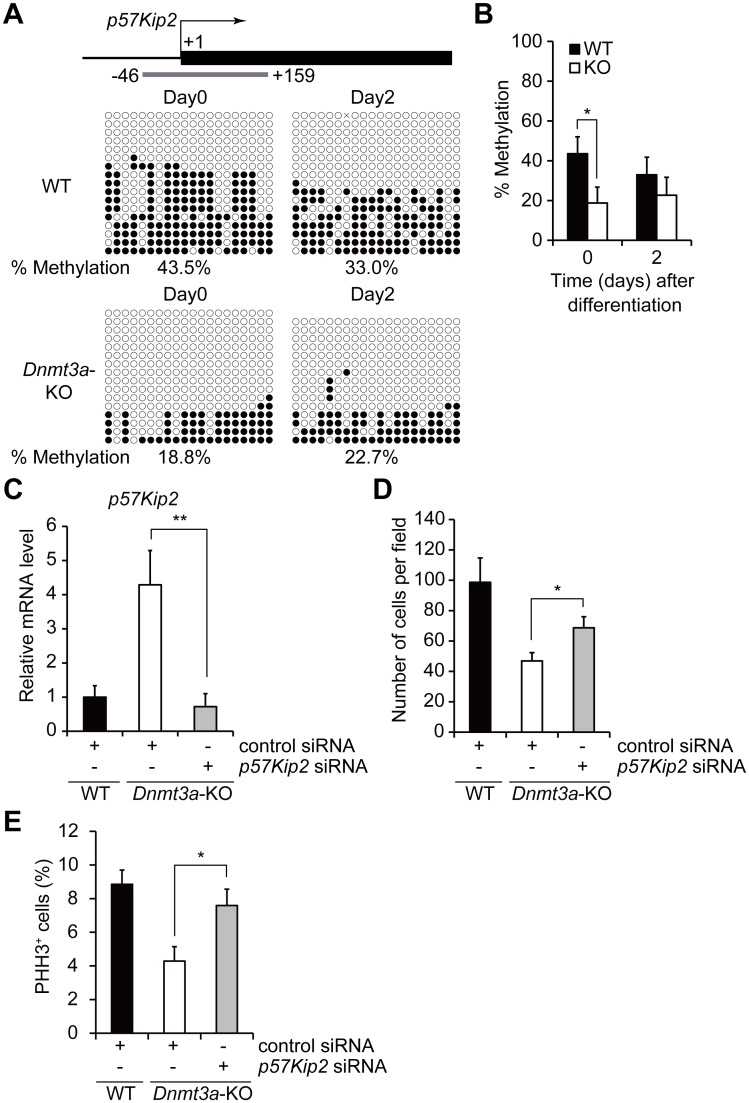
*p57Kip2* is a methylation target of Dnmt3a and regulates proliferation of SCs. (A) A schematic diagram of bisulfite sequencing at the *p57Kip2* locus of *Dnmt3a*-KO and WT SCs on day 0 and day 2 after inducing differentiation. White circles and black circles indicate unmethylated and methylated CpG sites, respectively. The transcription start site (TSS) is indicated by the bent arrow. The gray bar represents the sequencing target (+1 corresponds to the TSS). (B) Average methylation frequency of CpG sites at the *p57Kip2* locus; *p<0.05, Student’s t-test. SEMs between sequences are shown. (C) *p57Kip2* knockdown by siRNA in *Dnmt3a*-KO and WT SCs; *p<0.01 Student’s t-test. (D) Cell proliferation assay after *p57Kip2* knockdown. Numbers of SCs 3 days after siRNA transfection are shown. Reduced cell proliferation in *Dnmt3a*-KO SCs is partially rescued by *p57Kip2* knockdown; *p<0.05 Student’s t-test. Data represent mean ± SEM. (E) Quantification of PHH3 positive SCs with p57Kip2 knock-down: *p<0.05 Student’s t-test. Data represent mean ± SEM. The sequences of all primers used for PCR are listed in S1 Table.

To determine whether the mis-expression of *p57Kip2* in *Dnmt3a*-KO SCs is attributable to alteration of DNA methylation, we performed a bisulfite sequencing analysis in the *Pax7*-dependent *Dnmt3a*-KO and WT SCs. It was found that the *p57Kip2* promoter region was extremely hypomethylated in the undifferentiated *Dnmt3a*-KO SCs (Fig 5A and 5B), suggesting that the extent of DNA methylation in the promoter region underlies *p57Kip2* expression. Since we confirmed by lineage tracing that pure Pax7^+^ cells were isolated by the single myofiber culture method (S9 Fig), the difference in DNA methylation levels between *Dnmt3a*-KO and WT SCs did not seem to be due to contamination by non-myogenic cells. To examine whether *p57Kip2* is a functional target of Dnmt3a in regulating the proliferation of SCs, we tested the effect of *p57Kip2* depletion in the *Dnmt3a*-KO SCs. The cell proliferation defect was partially but significantly rescued by *p57Kip2* knockdown (Fig 5C and 5D). In line with these data, the decreased frequency of PHH3^+^ *Dnmt3a*-KO SCs was also partly rescued by *p57Kip2* knockdown (Fig 5E), indicating that *Dnmt3a* regulates the proliferation of SCs by controlling the expression of *p57Kip2*. Accordingly, our findings suggest that the decreased proliferation of SCs is, at least partly, due to mis-expression of *p57Kip2* caused by DNA hypomethylation. DNA hypomethylation of the *p57Kip2* promoter in the *Dnmt3a*-KO SCs prompted us to suppose that it is a methylation target of Dnmt3a. To assess the recruitment of Dnmt3a to the *p57Kip2* regulatory region, a ChIP-qPCR analysis was performed with Dnmt3a in undifferentiated proliferating WT SCs. The *p57Kip2* regulatory region was enriched with Dnmt3a at a similar level as the *H1foo* promoter, which is DNA-methylated except in oocytes (S10A Fig). The primers for the ChIP in the *H1foo* locus were designed on the basis of Dnmt3a2-ChIP-seq data by Baubec et.al [49] (S10B Fig). The housekeeping gene *Rps18* promoter, which is consistently DNA hypomethylated, was not enriched with Dnmt3a. These findings suggest that the *p57Kip2* regulatory region is a direct methylation target of Dnmt3a in SCs. In contrast to *p57Kip2*, the *p16INK4a* promoter region was not enriched in the Dnmt3a ChIP (S10A Fig), suggesting that this region is not a direct target of Dnmt3a. Taken together, *p57Kip2* is a methylation target of Dnmt3a and regulates proliferation of SCs.

### *In vivo* DNA hypomethylation and mis-expression of *p57Kip2* in *Dnmt3a*-cKO muscles

**Fig 6:**
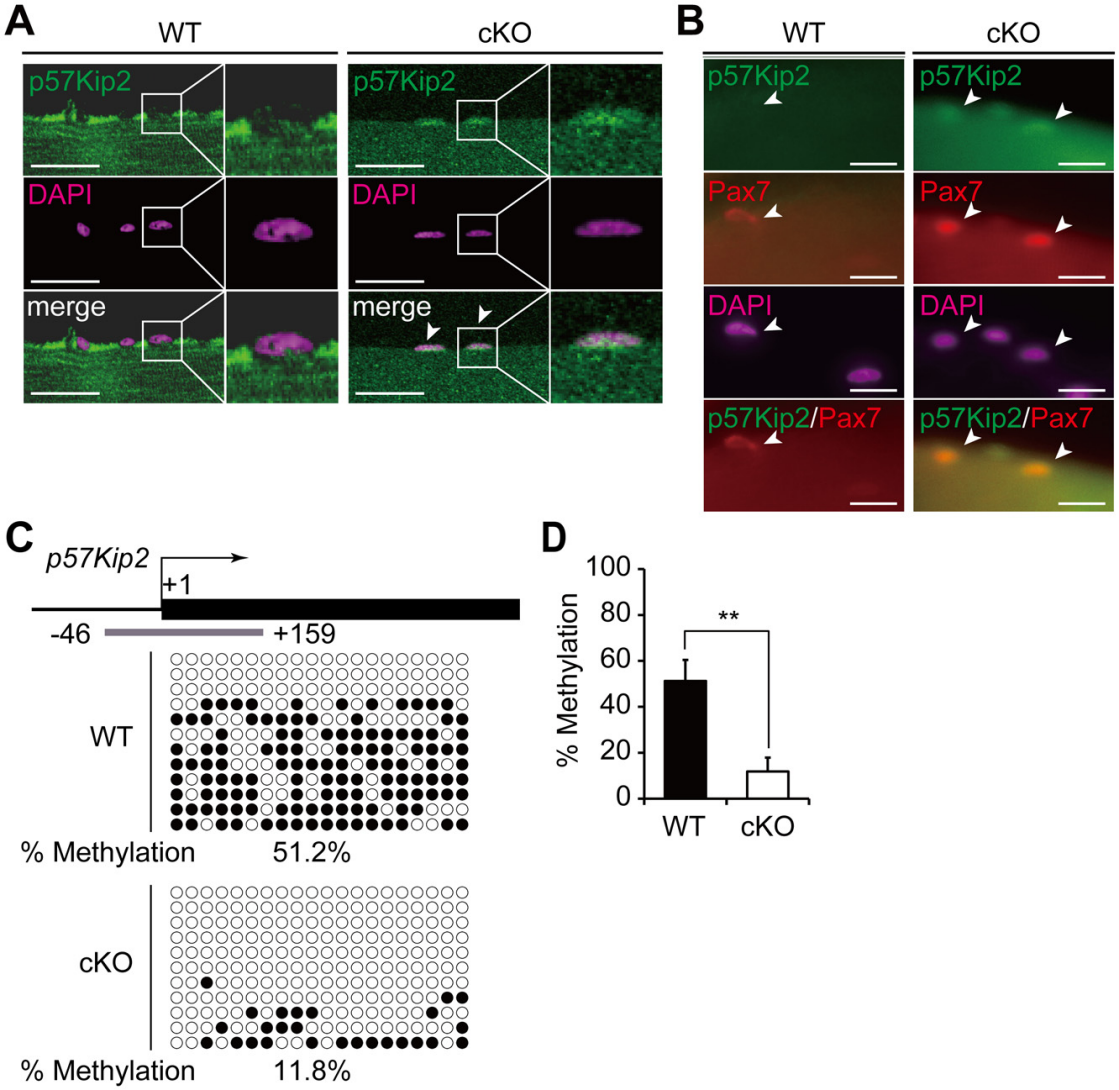
*In vivo* DNA hypomethylation and mis-expression of *p57Kip2* in *Dnmt3a*-cKO muscles. (A) Immunohistochemistry of p57Kip2 in *Dnmt3a*-cKO and WT single myofibers. Arrowheads indicate p57Kip2^+^ nuclei. Scale bar—30 μm. (B) p57Kip2/Pax7 co-staining in single myofibers. Arrowheads indicate Pax7^+^ nuclei. Scale bar—20 μm. (C) A schematic diagram of bisulfite sequencing at the *p57Kip2* locus in *Dnmt3a*-cKO and WT muscles. White circles and black circles indicate unmethylated and methylated CpG sites, respectively. The TSS is shown by the bent arrow. The gray bar represents the sequencing target (+1 corresponds to the TSS). (D) Average methylation frequency of CpG sites at the *p57Kip2* locus in *Dnmt3a*-cKO and WT muscles; *p<0.05, Student’s t-test. SEMs between sequences are shown.

To extend our *in vitro* findings to an *in vivo* context, we checked *p57Kip2* expression in the *Pax3-Cre; Dnmt3a-*cKO muscles. Immunostaining with a p57Kip2 antibody in single myofibers revealed a higher level of p57Kip2 protein expression in *Dnmt3a*-cKO muscles (Fig 6A). We further performed costaining of Pax7 and p57Kip2 in *Dnmt3a*-cKO and WT myofibers. The expression of p57Kip2 was very weak in the WT Pax7^+^ SCs (Fig 6B). In contrast, p57Kip2 was costained with Pax7 in the cKO myofibers, indicating that expression of p57Kip2 is indeed enhanced in the SCs (Fig 6B). Bisulfite sequencing analysis revealed significant hypomethylation at the promoter region of *p57Kip2* in the *Dnmt3a*-cKO muscles (Fig 6C and 6D), corroborating the findings in the *Pax7-Cre; Dnmt3a*-KO SCs. Since *p57Kip2* is also mis-expressed in the *Dnmt3a*-cKO muscles, this implies that Dnmt3a regulates *p57Kip2* expression through epigenetic mechanisms, both *in vitro* and *in vivo*.

### Proliferation of SCs *in vivo* during muscle regeneration is impaired in *Dnmt3a*-cKO mice

**Fig 7:**
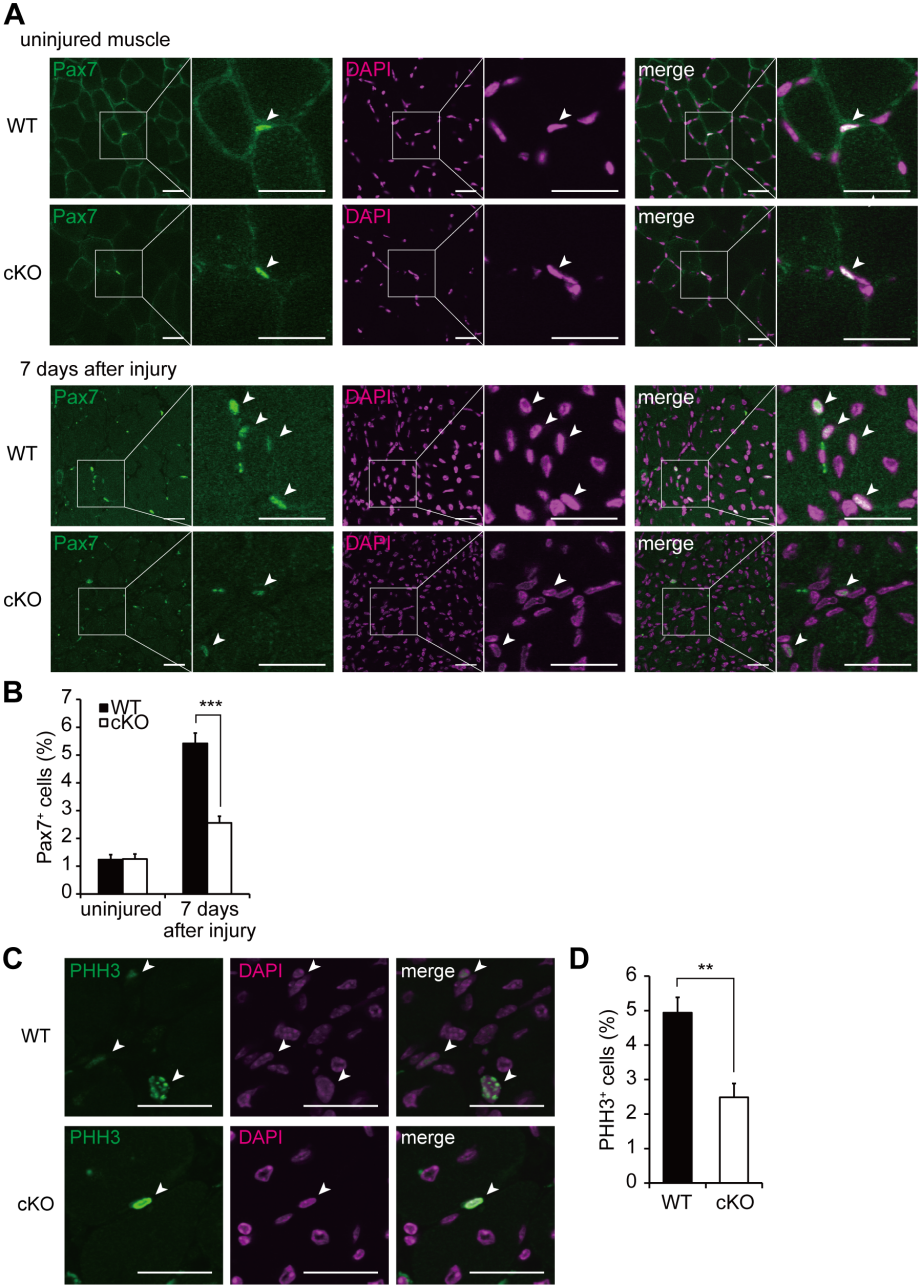
Proliferation of SCs *in vivo* during muscle regeneration is impaired in *Dnmt3a*-cKO mice. (A) Pax7 immunohistochemistry of the tibialis anterior muscle sections of *Dnmt3a*-cKO and WT mice. upper: uninjured muscle. lower: 7 days after injury. Arrowheads indicate Pax7^+^ nuclei. (B) Quantification of Pax7-expressing cells in the muscle sections 7 days after injury. (C) PHH3 immunohistochemistry of the tibialis anterior muscle sections of *Dnmt3a*-cKO and WT mice, 7 days after injury. Arrowheads indicate PHH3^+^ nuclei. (D) Quantification of PHH3^+^ cells in the muscle sections 7 days after injury; ***p<0.001, Student’s t-test. Scale bar—30 μm.

Our findings indicate that *Dnmt3a* loss impairs muscle regenerative capacity and reduces proliferative capacity of SCs. To determine whether the impaired muscle regeneration was a result of impaired SC proliferation, we assessed the frequency of SCs expressing Pax7 in both the unperturbed and the regenerating muscles. The frequency of Pax7^+^ cells in all nucleated cells in unperturbed *Pax3-Cre; Dnmt3a*-cKO muscles was not significantly different from that in WT muscles (Fig 7A and 7B). However, in the regenerating muscles, Pax7^+^ cells were less frequent in the *Dnmt3a*-cKO mice than in the WT mice (Fig 7A and 7B). Pax7/Laminin costaining demonstrated that most of these Pax7^+^ cells were located inside the basal lamina of the regenerated myofibers (S11 Fig). Next, to examine whether the lower frequency of Pax7^+^ cells in the *Dnmt3a*-cKO regenerating muscles was caused by decreased proliferation of the SCs, phospho-histone H3 (Ser10) immunostaining was performed in the regenerating tibialis anterior muscles. Immunostaining at 7 days after CTX injection revealed that PHH3^+^ cells were less frequent in the *Dnmt3a*-cKO than WT mice (Fig 7C and 7D). These results suggest that the SCs are not wasting in the uninjured muscles of *Dnmt3a*-cKO mice, but that their ability to proliferate after injury is impaired, leading to defects in their regenerative capacity. Immunostaining with a p57Kip2 antibody showed that p57Kip2^+^ cells were more frequent in the *Dnmt3a*-cKO than in the WT regenerating muscles (S11B and S11C Fig). The behavior of SCs was explored by Pax7/MyoD-costaining and Myog immunostaining in regenerating muscles. The ratios of MyoD^+^Pax7^+^ cells to MyoD^-^Pax7^+^ cells were lower in *Dnmt3a*-cKO regenerating muscles than in the WT (S11D and S11E Fig), suggesting SC activation is impaired in *Dnmt3a*-cKO muscles. Myog^+^ cells were less frequent in *Dnmt3a*-cKO regenerating muscles compared to those in the WT (S11F and S11G Fig). This lower frequency of Myog^+^ cells does not necessarily indicate impaired differentiation capacity as a result of the Dnmt3a deletion, because *Dnmt3a*-cKO reduced the number of proliferating SCs, which produce the differentiating SCs. Taken together, these results suggest that the SCs are not wasting in the uninjured muscles of *Dnmt3a*-cKO mice but their proliferation after injury is impaired, leading to the defects in the regenerative capacity.

In summary, Dnmt3a regulates the proliferation of muscle SCs, thereby influencing the growth of SCs in culture and the regenerative capacity of skeletal muscles. Hence, Dnmt3a maintains muscle homeostasis by regulating the functions of SCs through the epigenetic regulation of *p57Kip2*.

## Discussion

**Fig 8:**
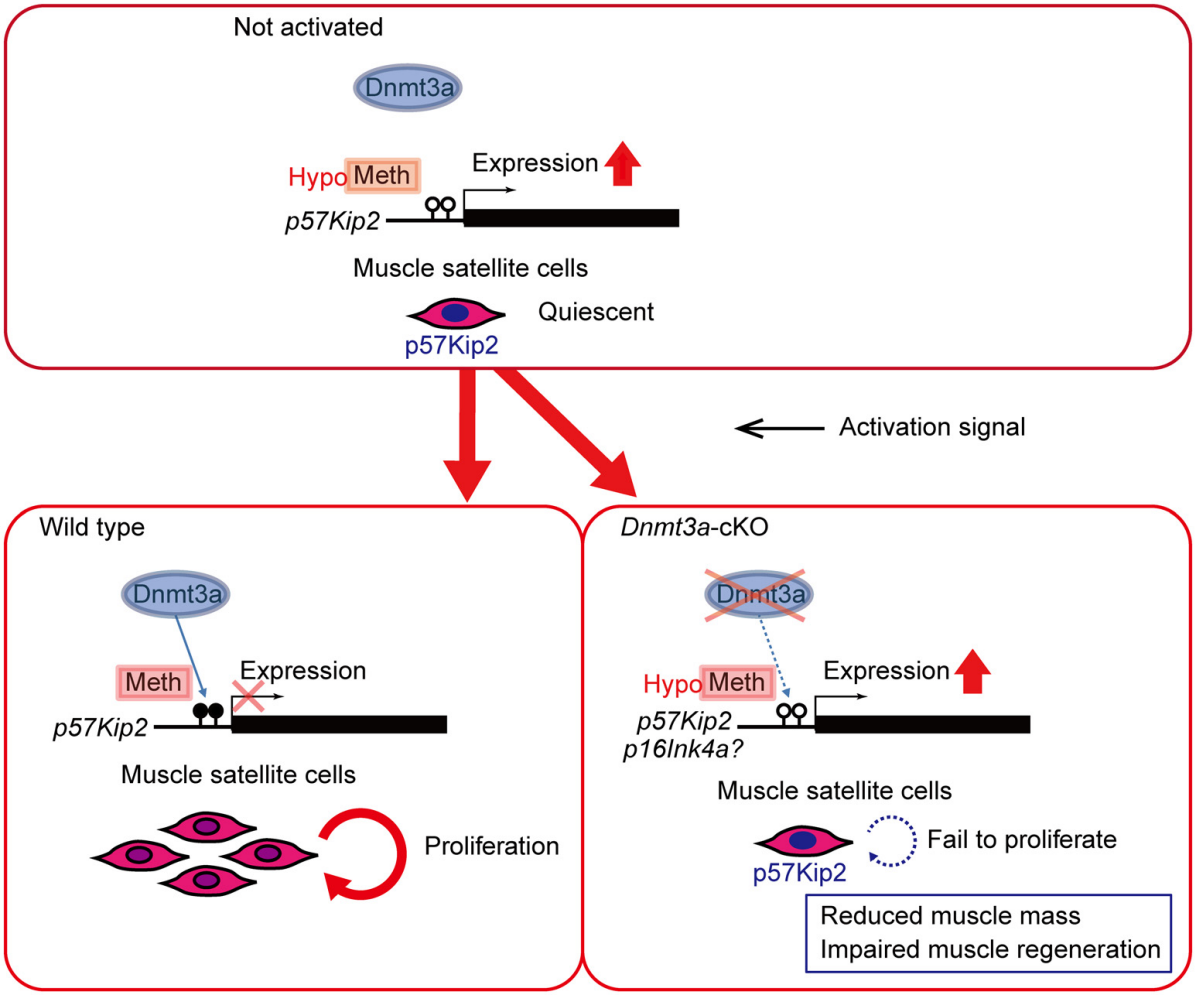
A schematic diagram of the *Dnmt3a-p57Kip2* axis in muscle satellite cells. *Dnmt3a* regulates proliferation of SCs via DNA methylation of the *p57Kip2* promoter. Loss of *Dnmt3a* leads to decreased proliferation of SCs, resulting in reduced muscle mass and impaired muscle regeneration.

In this study, we have shown that loss of *Dnmt3a* in the *Pax3*-expressing cell lineage leads to reduced body size and muscle mass in mice. Although *Pax3*-Cre; *Dnmt3a*-cKO mice exhibited grossly normal tissue patterns, they had thinner myofibers, unproportionally decreased muscle mass and impaired muscle regeneration, suggesting that Dnmt3a contributes to the function of SCs that are responsible for postnatal muscle growth and regeneration. *Pax7*^*-/-*^ mice which completely lack SCs display similar phenotypes to those of *Dnmt3a*-cKO mice, namely decreased muscle mass and reduced myofiber diameter, although the overall organization of myofibers appears normal [40]. The phenotypes of *Pax7*^*-/-*^ mice are attributable to a lack of SC fusion during the postnatal period [40]. We also identified *p57Kip2* as an essential downstream target of Dnmt3a for methylation and a causative candidate gene for the functional deficits in *Dnmt3a*-cKO SCs. This is corroborated by the finding that *p57Kip2* knockdown ameliorates the decreased proliferation of the *Dnmt3a*-cKO SCs. *Dnmt3a* deletion in SCs impairs proliferation through the mis-expression of *p57Kip2*, resulting in quantitative insufficiency of SCs similar to that in *Pax7*^*-/-*^ mice (Fig 8).

Roles of *p57Kip2* in regulating body and organ sizes have been elucidated in the context of human overgrowth and undergrowth disorders. *p57Kip2*-deficient mice have phenotypes similar to the manifestations of Beckwith-Wiedemann syndrome (BWS), an overgrowth disorder [50,51]; in addition, *p57Kip2* activity is lower in BWS patients [44,52]. Silver-Russell syndrome (SRS) is a heterogeneous disorder characterized by pre- and post-natal growth retardation [53,54]. IMAGe syndrome is another undergrowth disorder characterized by intrauterine growth retardation, metaphyseal dysplasia, adrenal hypoplasia and genital anomalies [55]. Loss-of-function mutations of *p57Kip2* have been identified in BWS patients [44], and gain-of-function mutations in the Proliferating cell nuclear antigen (PCNA)-binding domain of *p57Kip2* have been identified in growth retardation syndromes such as SRS and IMAGe syndrome [46–48].

It is well known that genomic imprinting is controlled by DNA methylation and that *p57Kip2* is paternally imprinted. DNA methylation at the imprint center is maintained by Dnmt1, a maintenance DNA methyltransferase, but Dnmt1 alone is not able to maintain all of the DNA methylation loci, especially in CpG-rich regions [24,56]. Therefore, there is a possibility that maintenance DNA methylation deficits besides *de novo* DNA methylation is caused by Dnmt3a deletion, resulting in the progressive loss of genomic imprinting. However, we think the mis-expression of *p57Kip2* in *Dnmt3a-*KO SCs is not a result of lost genomic imprinting because the imprint center is not located in the *p57Kip2* promoter and because *p57Kip2* is expressed only from the methylated maternal allele [52]. Considering this regulatory mechanism, the expression of *p57Kip2* should be decreased as a result of loss of genomic imprinting. In our *Dnmt3a*-KO SCs, *p57Kip2* expression level was lower than that of the WT, which implies that there was no change in genomic imprinting.

If the cell population is perfectly homogeneous, the DNA methylation level of a CpG site should be either 100% or 0%. Isolated SCs in our experiments are all Pax7-positive (S9 Fig), but their differentiation status after *in vitro* culture is not perfectly homogeneous. We consider some SCs might not get out of quiescence and others might be beginning spontaneous differentiation, and therefore the DNA methylation levels of WT SCs at the *p57Kip2* promoter were not 100%. In fact, during culture of isolated myofibers, some SCs divide asymmetrically into two types of cells that are distinctively fated to self-renew or to differentiate [57]. Hence, SCs are considered heterogeneous population composed of stem cells and committed progenitors. A certain proportion of SCs may divide asymmetrically even when cultured on dish. In addition, a DNA methylation level of the *p57Kip2* promoter was not 0% even in *Dnmt3a*-KO SCs. This might be because Dnmt3b incompletely compensates the influences of *Dnmt3a* deletion.

Although our findings reveal an essential role of *p57Kip2* in the undifferentiated SCs, *p57Kip2* is also known to be a target of MyoD, which promotes muscle differentiation [58]. We also observed a further increase in the expression of *p57Kip2* after myogenic differentiation, coincident with the cell cycle deceleration in the differentiating SCs. Our findings suggest that *Dnmt3a*-KO prematurely triggers the induction of *p57Kip2* in the undifferentiated SCs, which results in a reduced number of SCs forming mature myofibers.

The decrease in body size and muscle mass of *Dnmt3a*–cKO mice were more severe in females. We could not identify the reason for this gender difference; previous studies of *Dnmt3a* deletion in other tissues have not reported such gender-dependent severity of phenotypes. However, female mice show more severe phenotypes of several heart diseases [59,60]. In the mdx mouse model of Duchenne cardiomyopathy, aged female mice display more severe cardiomyopathy [61]. Although the detailed reasons for such differences are not clear, it is possible that the female muscular tissues are more susceptible to a specific pathological condition.

Another epigenetic regulatory mechanism, histone modification is also known to regulate SC functions. Histone deacetylase inhibitors increase muscle cell size by promoting cell fusion without affecting cell proliferation [62]. On the other hand, conditional ablation of Polycomb-repressive complex (PRC2) subunit EZH2 in Pax7^+^ cells results in impaired SC proliferation and reduced muscle mass with small myofibers [63]. Taken together, it is suggested that multiple epigenetic mechanisms coordinately regulate SC functions and control the tissue size of skeletal muscles.

Thus, the loss of *Dnmt3a* in muscle progenitor cells leads to premature expression of a CDKI, *p57Kip2*, which causes decreased proliferation of the SCs, leading to smaller body size and disproportionately reduced muscle mass in mice. Our findings indicate that there are several potential mechanisms for size regulation. Firstly, DNA methylation, which specifies the sets of genes to be expressed in a certain context, influences body size. Secondly, the number of tissue stem cells, which is balanced between self-renewal and differentiation commitment, might influence body and organ sizes. There is an increased incidence of rhabdomyosarcoma among BWS patients [64,65], which implies that deteriorated size regulation leads to tumorigenesis. Our current understanding of the mechanisms regulating body and organ size is limited; however, further elucidation of the size control machinery may lead to novel therapeutic approaches for cancer that target these mechanisms.

In this study, we show that *Dnmt3a* regulates proliferation of muscle SCs by methylating the *p57Kip2* locus and suggest that this *Dnmt3a-p57Kip2* axis forms the basis of size-control mechanisms in muscle tissues. Further elucidation of the underlying relation between DNA methylation and body and organ size control, will provide novel insights for developing new therapeutic approaches for some of the incurable human disorders.

## Materials and Methods

### Ethics statement

We used mice in our research. The mice were anesthetized by intraperitoneal injection of pentobarbital or inhalation of isoflurane. Cervical dislocation was used as a euthanasia method. All animal experiments were approved by the Institutional Animal Care and Use Committee at Tokyo Medical and Dental University (approval number; 0160127A).

### Mice

*Dnmt3a*-flox mice were kindly provided by Dr. M. Okano. *Dnmt3a*-floxed allele was previously described [66]. *Pax3*-Cre mice and *Pax7*-CreERT2 mice were purchased from the Jackson Laboratory (Bar Harbor, ME). *Pax3*-Cre allele and *Pax7*-CreERT2 allele were previously described [67,68]. Genomic DNA was isolated from muscle tissues using DNeasy Blood & Tissue Kit (Qiagen, Hilden, Germany) according to the manufacturer’s instructions. Gene deletion efficiency was calculated by genomic DNA qPCR. Relative genomic DNA level was determined by the standard curve method. All primer sequences are listed in S1 Table.

### Computed tomography scan

Computed tomography (CT) scan of distal hindlimbs was performed using Latheta LCT-200 (Hitachi Aloka Medical, Tokyo, Japan). Mice were anesthetized by isoflurane inhalation during the scan. The image data were analyzed using Latheta software (Hitachi Aloka Medical, Tokyo, Japan), and muscle and bone cross-sectional volume were calculated. The slice of each limb where the muscle cross-sectional area was the greatest was selected for muscle volume evaluation, for each mouse.

### Histological analysis

Muscle tissues of 8- to 12week-old mice were frozen in isopentane cooled in liquid nitrogen. Frozen tissues were sectioned using a cryostat CM3050S (Leica, Wetzlar, Germany) at 10 μm thickness and mounted on MAS-coated slide glasses (Matsunami Glass, Osaka, Japan). The CSA of myofibers were measured in at least five fields of view using ImageJ software (National Institutes of Health, Bethesda, MD). For Hematoxylin-Eosin (HE) staining, muscle sections were fixed in 4% paraformaldehyde (PFA) in phosphate buffered saline (PBS) at room temperature for 10 minutes, then immersed in Mayer’s Hematoxylin Solution (Wako, Osaka, Japan) for 5 minutes, followed by washing under running water for 10 minutes. After staining with 1% Eosin Y Solution (Wako, Osaka, Japan) for 1 minute, they were sequentially immersed in 70%, 95% and 100% ethanol for 30 seconds, 1 minute and 3 minutes, respectively. Finally, they were washed thrice in xylene for 3 minutes each and embedded in Entellan Neu (Merck KGaA, Darmstadt, Germany).

### Culture of satellite cells

*Dnmt3a-*KO SCs were harvested from 6- to 8-week-old *Pax7-CreERT2; Dnmt3a*^*flox/flox*^ mice. Tamoxifen (Sigma, St Louis, LA) was administered to the mice intraperitoneally at the dose of 100 μg/body weight (g) for 5 consecutive days. After seven days of the first tamoxifen administration, the mice were sacrificed to harvest gastrocnemius muscles, and SCs were isolated as previously described [69,70]. Briefly, single myofibers were obtained by collagenase digestion and cultured in primary cultured myocyte growth medium (pmGM) consisting of Dulbecco’s modified Eagle’s medium (DMEM; Sigma, St Louis, LA) with 20% fetal bovine serum, 1% penicillin/streptomycin (Life Technologies, Grand island, NY), 2% Ultroser G (Pall, New York, NY), 1000 U/ml mouse leucocyte inhibitory factor (LIF; AMRAD Biotech, Victoria, Australia) and 10 ng/ml human basic fibroblast growth factor (bFGF; PeproTech EC, London, UK) on type I collagen-coated dishes (Sumilon, Tokyo, Japan) at 37°C under 10% CO_2_ in a humidified chamber. SCs migrated from the myofibers in 4 to 5 days. For analyzing growth of SCs, isolated SCs were cultured in pmGM. To induce myogenic differentiation, SCs were cultured in DMEM with 2% horse serum.

### Immunostaining

Frozen muscles were sectioned at 10 μm thickness and mounted on MAS-coated slide glasses (Matsunami Glass, Osaka, Japan). Single myofibers were isolated by collagenase digestion as previously described [69,70], and plated on MAS-coated slide glasses (Matsunami Glass, Osaka, Japan). Sections or myofibers were dried in the air and then fixed in 4% PFA in PBS at room temperature for 10 min. For immunocytochemistry, cultured cells are fixed in 4% PFA in PBS at room temperature for 10 min. After permeabilization with 0.1% Triton X-100 in PBS for 20 min, they were blocked with 1% Bovine serum albumin (BSA) in PBS for 1 hour and incubated with primary antibodies at 4°C overnight. The following antibodies were used: anti-Pax7 (described previously [71]), anti-MyoD (BD Pharmingen 554130, 1:100), anti-Myog (Santa Cruz sc-576, 1:50), anti-Phospho-Histone H3 (Ser10) (Cell Signaling #9701, 1:400), anti-active Caspase-3 (Abcam ab2302,1:200), anti-p57Kip2 (Santa Cruz sc-8298, 1:100), anti-p57Kip2 (Cell Signaling #2557, 1:500) and anti-Laminin 2 alpha (Abcam ab11576, 1:500). After the primary antibody incubation, sections were incubated with secondary antibodies conjugated with Alexa Fluor 488 or 594 (Life Technologies, 1:1000). Finally, they were mounted in VectaShield with DAPI (Vector Laboratories, CA, USA). The mean intensity of fluorescence signals in each cell was calculated using ImageJ software (National Institutes of Health, Bethesda, MD).

### EdU incorporation assay

SCs were harvested as described above and cultured in pmGM for about 7 days to expand enough for the assay. One day after a passage to adjust confluency, they were cultured in medium containing 10 μM EdU for 3 hours for EdU labeling. EdU incorporation was assessed using Click-iT Plus EdU Alexa Fluor 488 Imaging Kit (Life Technologies, Grand island, NY).

### Gene expression analysis

Total RNA was isolated from the homogenized muscle tissues using ISOGEN (Nippon Gene, Tokyo, Japan) according to the manufacturer’s instructions. One μg of total RNA was used to synthesize cDNA. Reverse transcription was performed using ReverTra Ace (Toyobo, Osaka, Japan) following the manufacturer’s instructions. qPCR was performed by Thermal Cycler Dice Real Time System II (Takara Bio, Japan) using Thunderbird SYBR qPCR Mix (Toyobo, Osaka, Japan) and the relative expression levels were detected by the ΔΔCt method. All primer sequences are listed in S1 Table.

Microarray analysis (Affymetrix) was performed with RNA samples derived from the WT- and *Dnmt3a*^*flox/flox*^-SCs infected with Ax-Cre (MOI 30) at 0, 12, 24, 48, 72 and 96 hours of differentiation *in vitro*. The data were normalized and z transformed for the hierarchical clustering analysis utilizing Multiple Experiment Viewer [72].

### Bisulfite sequencing

Bisulfite conversion of the isolated genomic DNA was performed by CpGenome Turbo Bisulfite Modification Kit (Millipore, Billerica, MA) according to the manufacturer’s instructions. Bisulfite-treated DNA was amplified by PCR using Quick Taq HS DyeMix (Toyobo, Osaka, Japan). All primer sequences are listed in S1 Table. PCR products were cloned into T-Vector pMD20 (Takara Bio, Shiga, Japan) and sequenced with the M13 reverse primer from at least 12 clones.

### Muscle injury and regeneration

Fifty microliters of 0.03 mg/ml cardiotoxin (CTX; Sigma, St Louis, LA) was injected into the bilateral tibialis anterior muscles of 8- to 12-week-old mice, after making skin incisions to expose the fascia on bilateral hindlimbs under anesthesia. The mice were sacrificed 7 to 14 days after CTX injection, and the injured muscles were harvested for histological analysis and gene expression analysis.

### *p57Kip2* knockdown

*p57Kip2* knockdown was achieved by *p57Kip2* siRNA transfection. SCs were disseminated on type I collagen-coated dishes at a density of 0.1 × 10^5^ cells/ml. After verifying cell adherence to the dishes, siRNA was transfected at a final concentration of 20 nM, using Lipofectamine RNAiMAX Transfection Reagent (Invitrogen, Carlsbad, CA) according to the manufacturer’s instructions. SCs were counted daily, starting from day 1 after transfection. MISSION siRNA targeting murine *p57Kip2* was supplied by Sigma-Aldrich (St. Louis, MO). *p57Kip2* siRNA duplexes of the following RNA sequences were used: 5’-GUGCUGAGCCGGGUGAUGATT-3’; 5’-UCAUCACCCGGCUCAGCACTT-3’. AllStars Negative Control siRNA (Qiagen, Hilden, Germany) was used for the mock transfection control.

### Chromatin immunoprecipitation

Approximately 1.0 × 10^7^ proliferating SCs for each antibody were fixed with 1% formaldehyde at room temperature for 10 minutes. The cell lysates were sonicated with a Covaris S2 sonicator to shear DNA. Dynabeads Protein A (Invitrogen, Carlsbad, CA) conjugated with 10 μg of each primary antibody was added, followed by incubation at 4°C overnight. The beads were washed 5 times with RIPA buffer (0.2% NP-40, 0.2% Na-deoxycholate, 0.16 M LiCl, 10 mM EDTA, 20 mM HEPES-KOH, pH 7.6) and eluted with elution buffer (1% SDS, 50 mM EDTA, 100 mM Tris-HCl, pH 8.0). The eluate was incubated at 65°C overnight to reverse the crosslinking, followed by incubation at 55°C for 1 hour in the presence of proteinase K. DNA was purified using a MinElute PCR Purification Kit (Qiagen, Hilden, Germany) and quantified by real-time PCR (Thermal Cycler Dice Real Time System II (Takara Bio, Japan)). All primer sequences are listed in S1 Table.

## Supporting Information

S1 Fig Muscle manifestations of *Pax3-Cre; Dnmt3a*-cKO mice. (A) RT-qPCR analysis of *Dnmt3b* in the muscles of *Pax3*-Cre*; Dnmt3a*-cKO and WT mice. (B) Hindlimb muscles of *Pax3*-Cre*; Dnmt3a*-cKO mice are hypoplastic. (C) RT-qPCR analysis of myogenic gene expression in the muscles of *Pax3*-Cre*; Dnmt3a*-cKO and WT mice. No statistically significant difference between *Dnmt3a*-KO and WT muscles was detected.; Data represent mean ± SEM.
(TIF)

S2 Fig Proliferation capacity of *Pax3-Cre; Dnmt3a*-KO SCs. (A) Representative phase-contrast microscopic images of *Pax3-Cre; Dnmt3a*-KO and WT SCs. Both KO and WT cells were disseminated at the same cell density on Day 0. (B) *In vitro* cell proliferation assay shows significantly reduced proliferation of *Pax3-Cre;Dnmt3a*-KO SCs compared to WT SCs; ***p<0.001, two-way repeated measures ANOVA. Data represent mean ± SEM.
(TIF)

S3 Fig *Dnmt3a* expression levels in neonatal and adult SCs. RT-qPCR analysis of *Dnmt3a* and *MyoD* in neonatal and adult SCs. *Dnmt3a* expression level is higher in neonatal SCs than in adult SCs. No remarkable change of *Dnmt3a* expression is observed during muscle differentiation.
(TIF)

S4 Fig EdU incorporation assay in *Pax7-Cre; Dnmt3a-*KO SCs. (A) Representative images of fluorescent photomicrograph of *Dnmt3a*-KO and WT SCs after EdU administration. Arrowheads indicate EdU^+^ cells. Scale bar—30 μm. (B) Quantification of EdU^+^ cells in *Dnmt3a*-KO and WT SCs; ***p<0.001, Student’s t-test. Data represent mean ± SEM.
(TIF)

S5 Fig Apoptotic activity of *Pax7-Cre; Dnmt3a* SCs. Representative photomicrographs of *Pax7-Cre; Dnmt3a*-KO and WT SCs stained with cleaved Caspase 3 and DAPI. The frequency of cleaved Caspase-3-positivity was very low in both *Pax7-Cre; Dnmt3a*-KO and WT SCs. Scale bar—30 μm.
(TIF)

S6 Fig Differentiation capacity of *Pax7-Cre; Dnmt3a* SCs. (A) Morphologies of *Pax7-Cre; Dnmt3a*-KO and WT SCs differentiated *in vitro*. Phase-contrast micrograms of SCs—0, 1, 2 and 4 days after differentiation induction are shown. Scale bar—200 μm. (B) RT-qPCR analysis of myogenic gene expression in *Pax7-Cre; Dnmt3a*-KO and WT SCs. Data represent mean ± SEM.
(TIF)

S7 Fig Transcriptome analysis of AxCre-mediated *Dnmt3a*-KO SCs differentiated *in vitro*. (A) RT-qPCR analysis of myogenic genes in Ax-Cre; *Dnmt3a*-KO and WT SCs during differentiation. (B) A heat map of the gene expression profile showing the result of microarray analysis of WT SCs differentiated *in vitro*.
(TIF)

S8 Fig RT-qPCR analysis of CDKIs in *Pax7-Cre; Dnmt3a* SCs differentiated in vitro. RT-qPCR analysis of CDKIs in *Pax7-Cre; Dnmt3a*-KO and WT SCs during differentiation. *Dnmt3a*-KO SCs express *p16INK4a* at higher level than WT SCs.
(TIF)

S9 Fig Purity of isolated Pax7^+^ cells. Single myofibers were harvested from *Pax7-CreERT2; Rosa26R TdTomato* mice after tamoxifen administration. All the cells that have migrated from the myofibers are RFP positive, Scale bar—200 μm.
(TIF)

S10 Fig ChIP-qPCR analysis with Dnmt3a. (A) Enrichment of p57Kip2 promoter region is as high as H1foo promoter region. Two different pairs of primers are used for the *p57Kip2* promoter region. All primer sequences are listed in S1 Table. (B) Reanalysis of Dnmt3a2-ChIP-seq (GSE57413). Dnmt3a2-ChIP-seq data in ES cells. Results around the H1foo locus is shown. The primers for the ChIP in the H1foo locus were designed on the basis of Dnmt3a2-ChIP-seq data by Baubec et.al [49].
(TIF)

S11 Fig Immunostaining in regenerating *Pax3-Cre; Dnmt3a*-cKO and WT muscles. (A) Pax7/Laminin co-staining. Pax7^+^ cells are located inside the basal lamina in the regenerating myofibers, representing that SCs are stained by an anti-Pax7 antibody. Arrowheads indicate Pax7^+^ nuclei. Scale bar—20 μm. (B) p57Kip2 immunostaining. Arrowheads indicate p57Kip2^+^ nuclei. Scale bar—20 μm. (C) Quantification of p57Kip2^+^ cells; *p<0.05, Student’s t-test. (D) MyoD/Pax7 co-staining. Scale bar—10 μm. (E) Quantification of MyoD^+^Pax7^+^ cells. The ratios of MyoD^+^/Pax7^+^ cells to a total number of Pax7^+^ cells in each field of view are shown; *p<0.05, Student’s t-test. (F) Myog immunostaining. Scale bar—10 μm. (G) Quantification of Myog^+^ cells; **p<0.01, Student’s t-test.
(TIF)

S1 Table Primer sequences. (TIF)
