# Supplementary figures and images for: Dnmt3a Regulates Proliferation of Muscle Satellite Cells via p57Kip2
Source: PLoS Genet. 2016 Jul 14;12(7):e1006167. doi: 10.1371/journal.pgen.1006167 (PMC4944932; doi:10.1371/journal.pgen.1006167)

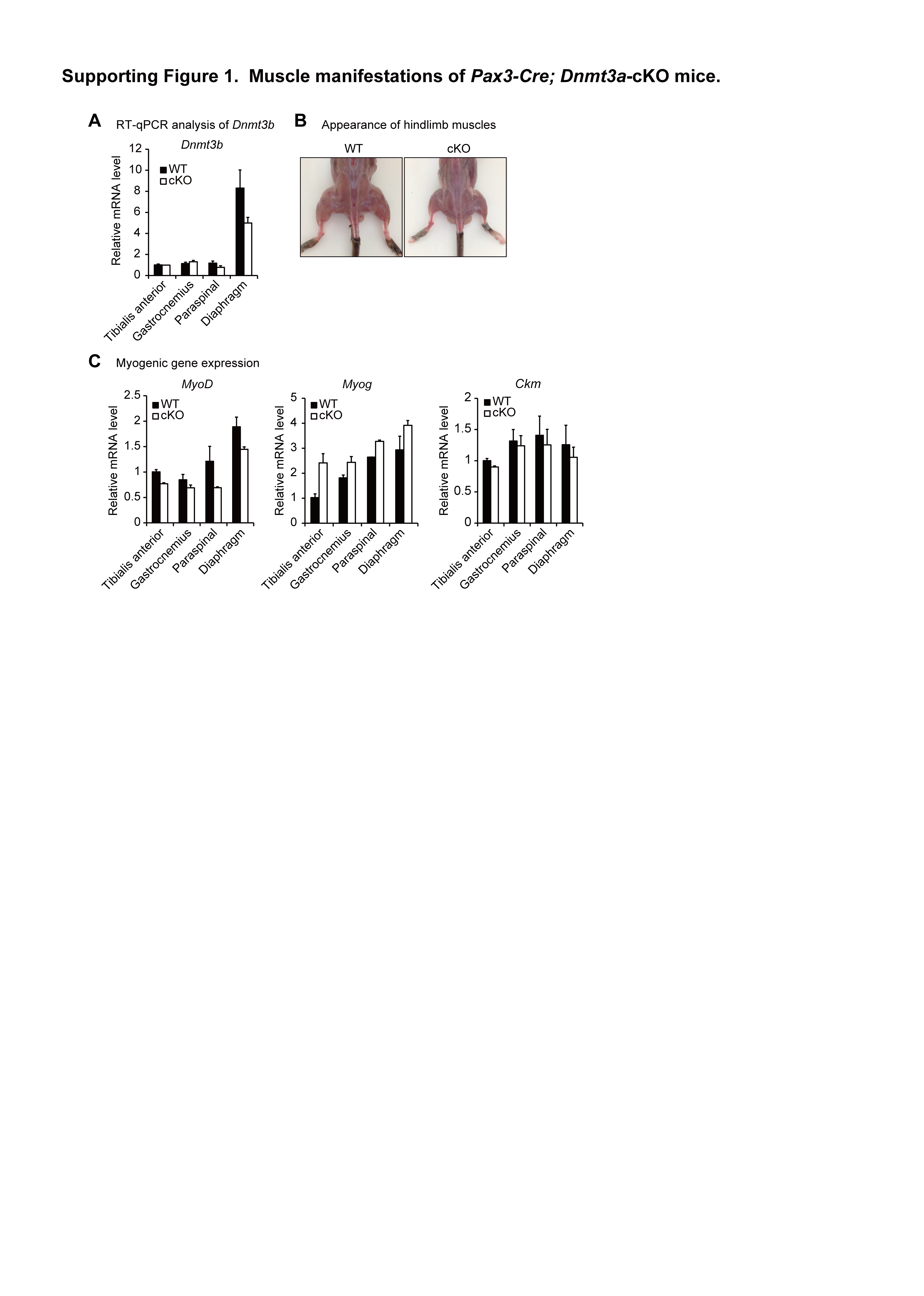

Supplement: S1 Fig — (A) RT-qPCR analysis of Dnmt3b in the muscles of Pax3-Cre; Dnmt3a-cKO and WT mice. (B) Hindlimb muscles of Pax3-Cre; Dnmt3a-cKO mice are hypoplastic. (C) RT-qPCR analysis of myogenic gene expression in the muscles of Pax3-Cre; Dnmt3a-cKO and WT mice. No statistically significant difference between Dnmt3a-KO and WT muscles was detected.; Data represent mean ± SEM. (TIF) [file pgen.1006167.s001.tif]

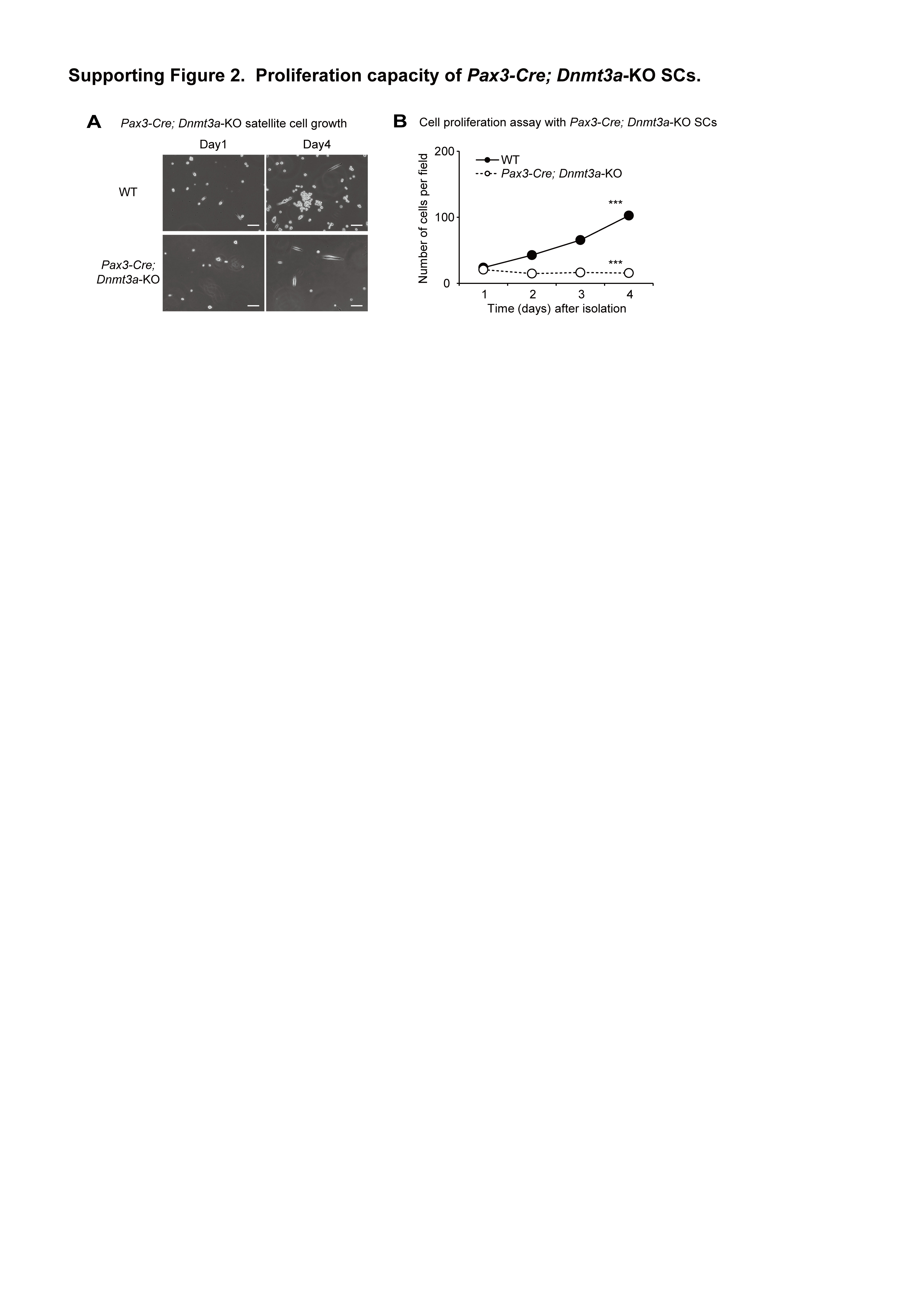

Supplement: S2 Fig — (A) Representative phase-contrast microscopic images of Pax3-Cre; Dnmt3a-KO and WT SCs. Both KO and WT cells were disseminated at the same cell density on Day 0. (B) In vitro cell proliferation assay shows significantly reduced proliferation of Pax3-Cre;Dnmt3a-KO SCs compared to WT SCs; ***p<0.001, two-way repeated measures ANOVA. Data represent mean ± SEM. (TIF) [file pgen.1006167.s002.tif]

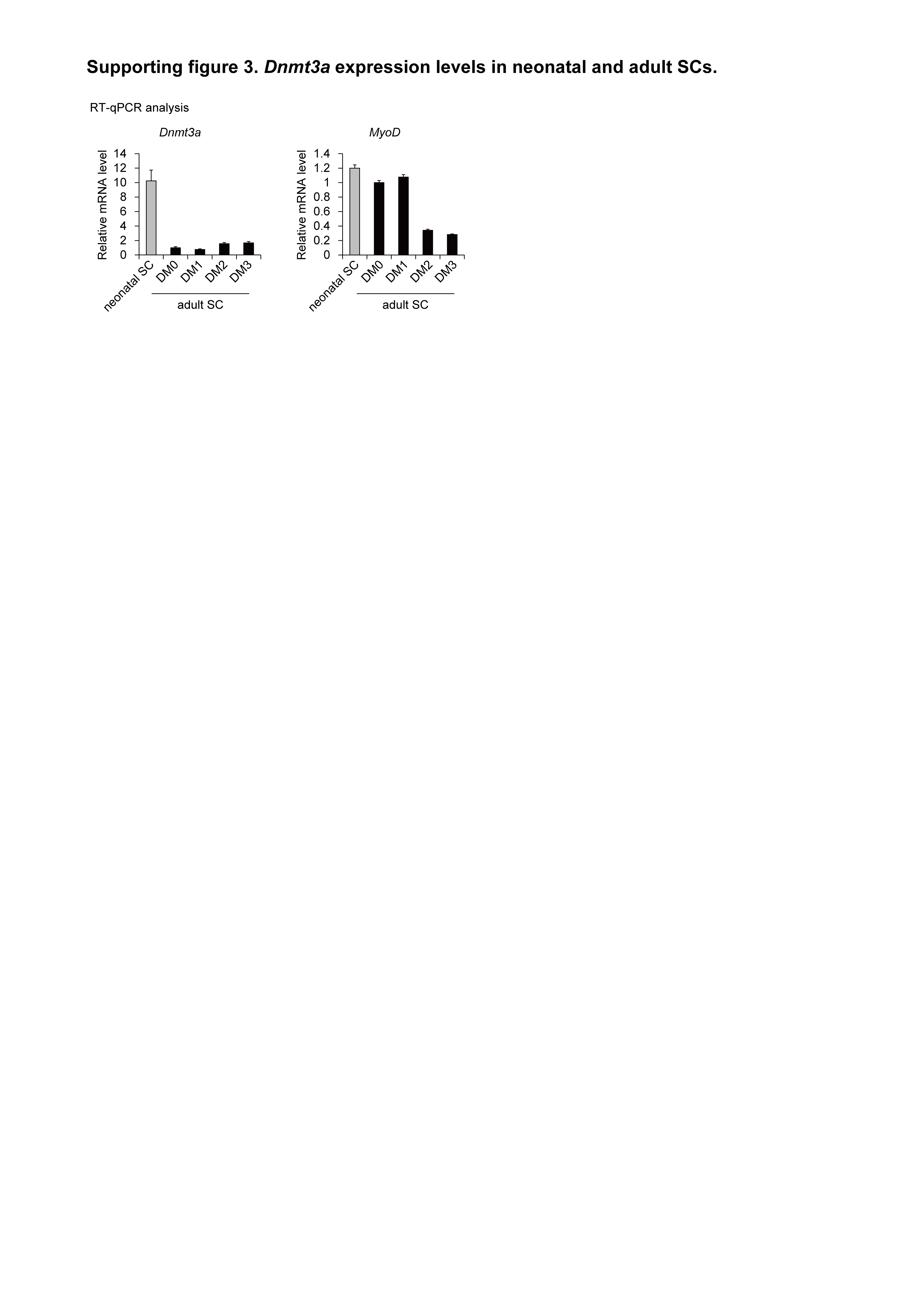

Supplement: S3 Fig — RT-qPCR analysis of Dnmt3a and MyoD in neonatal and adult SCs. Dnmt3a expression level is higher in neonatal SCs than in adult SCs. No remarkable change of Dnmt3a expression is observed during muscle differentiation. (TIF) [file pgen.1006167.s003.tif]

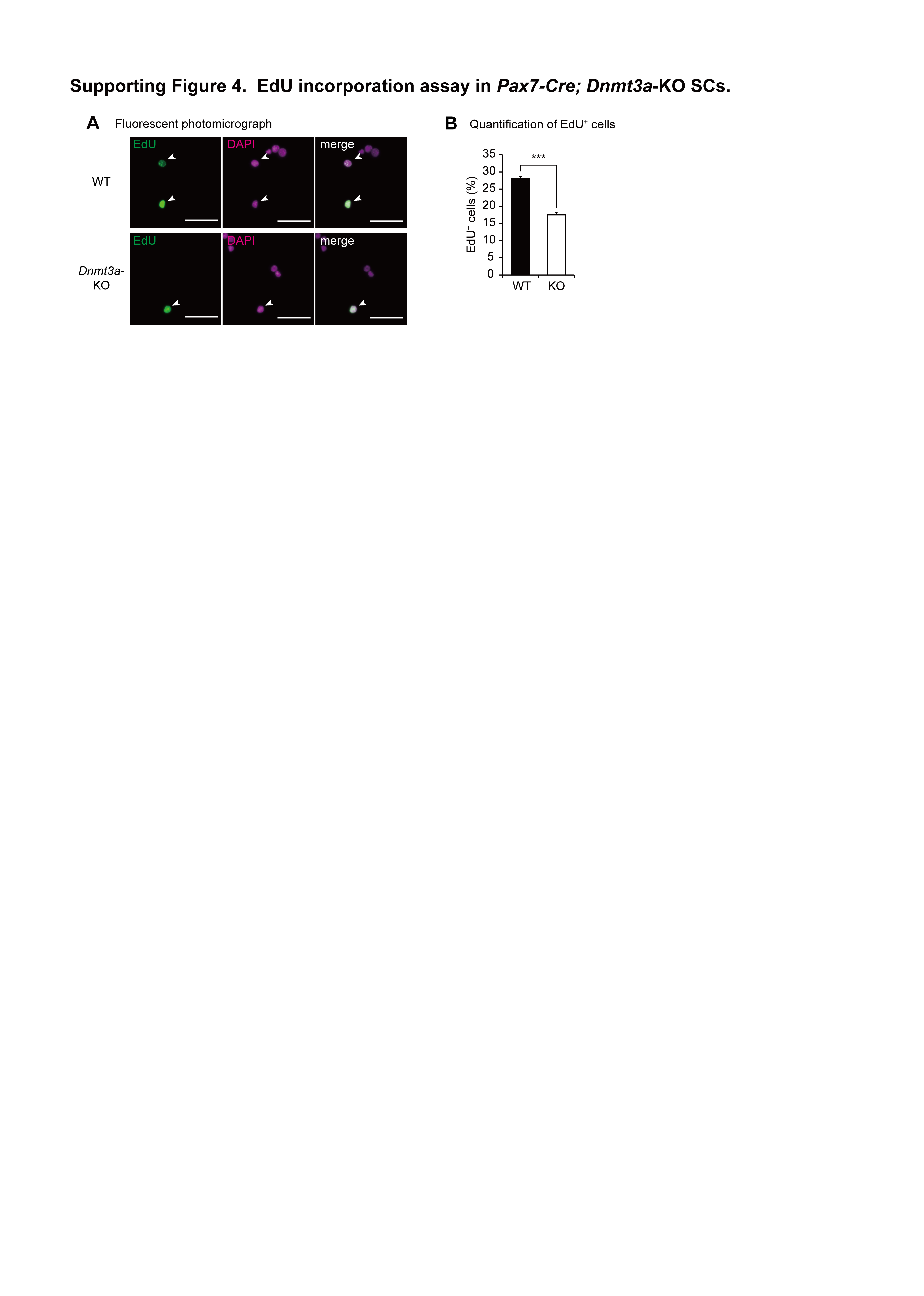

Supplement: S4 Fig — (A) Representative images of fluorescent photomicrograph of Dnmt3a-KO and WT SCs after EdU administration. Arrowheads indicate EdU+ cells. Scale bar—30 μm. (B) Quantification of EdU+ cells in Dnmt3a-KO and WT SCs; ***p<0.001, Student’s t-test. Data represent mean ± SEM. (TIF) [file pgen.1006167.s004.tif]

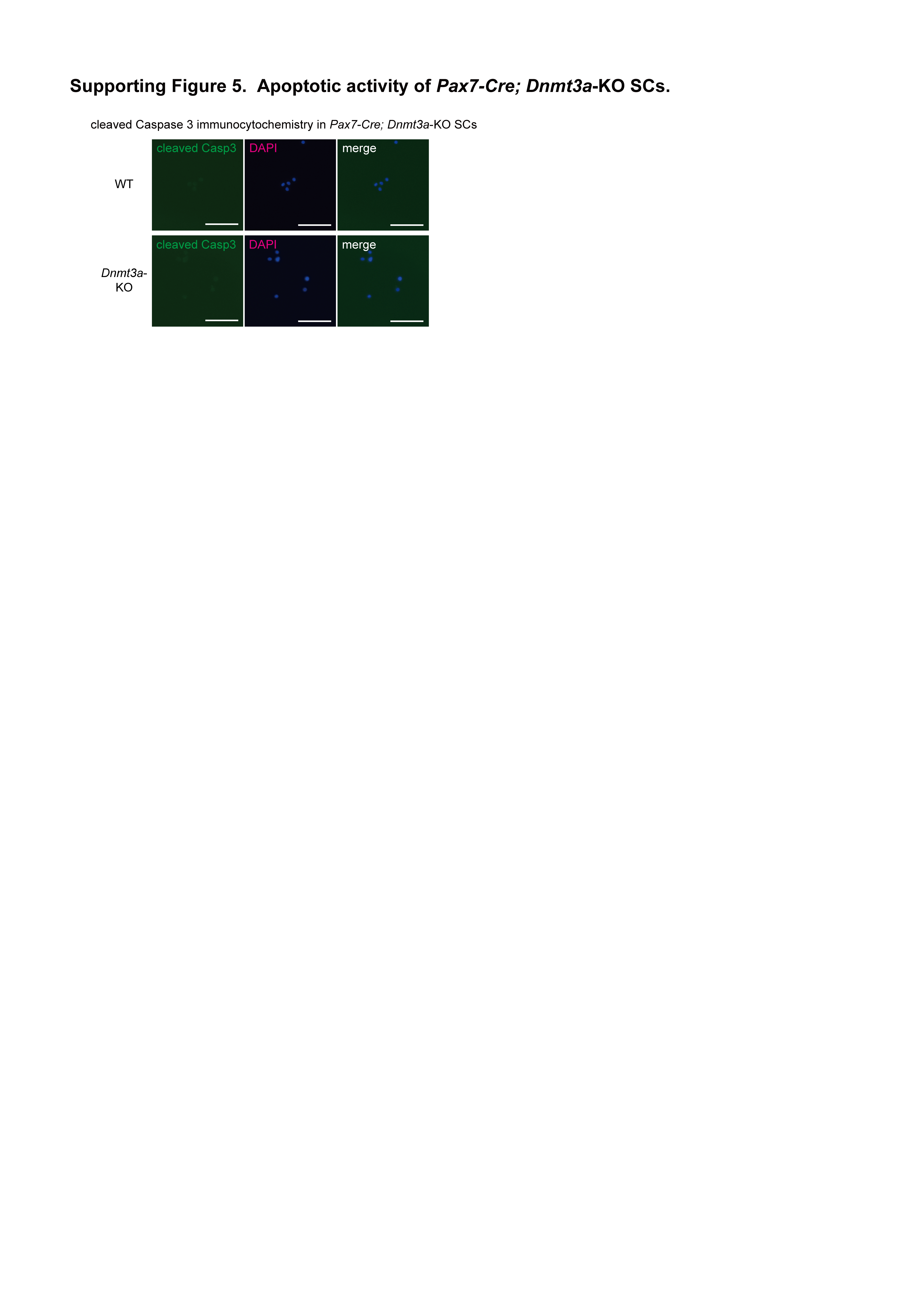

Supplement: S5 Fig — Representative photomicrographs of Pax7-Cre; Dnmt3a-KO and WT SCs stained with cleaved Caspase 3 and DAPI. The frequency of cleaved Caspase-3-positivity was very low in both Pax7-Cre; Dnmt3a-KO and WT SCs. Scale bar—30 μm. (TIF) [file pgen.1006167.s005.tif]

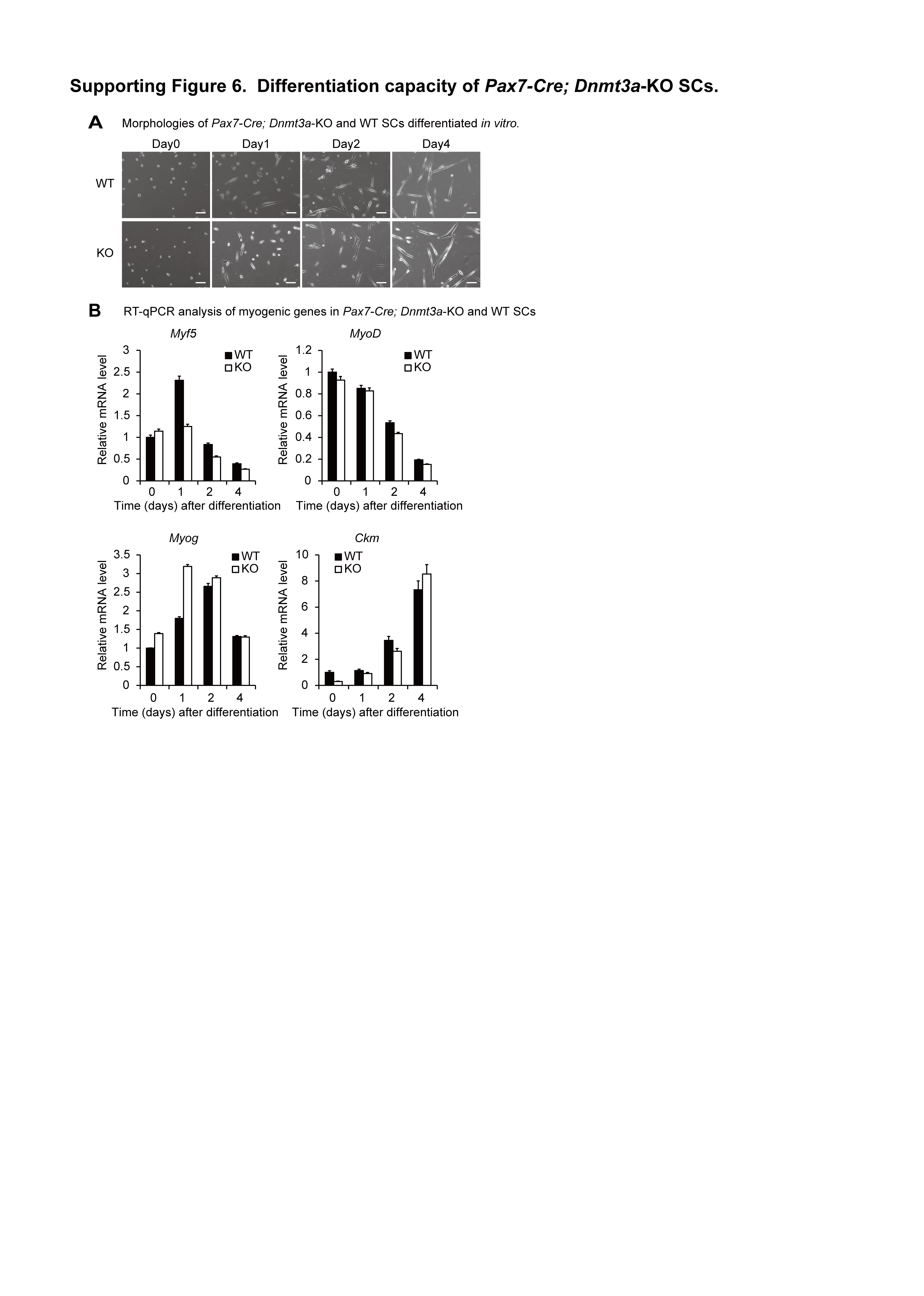

Supplement: S6 Fig — (A) Morphologies of Pax7-Cre; Dnmt3a-KO and WT SCs differentiated in vitro. Phase-contrast micrograms of SCs—0, 1, 2 and 4 days after differentiation induction are shown. Scale bar—200 μm. (B) RT-qPCR analysis of myogenic gene expression in Pax7-Cre; Dnmt3a-KO and WT SCs. Data represent mean ± SEM. (TIF) [file pgen.1006167.s006.tif]

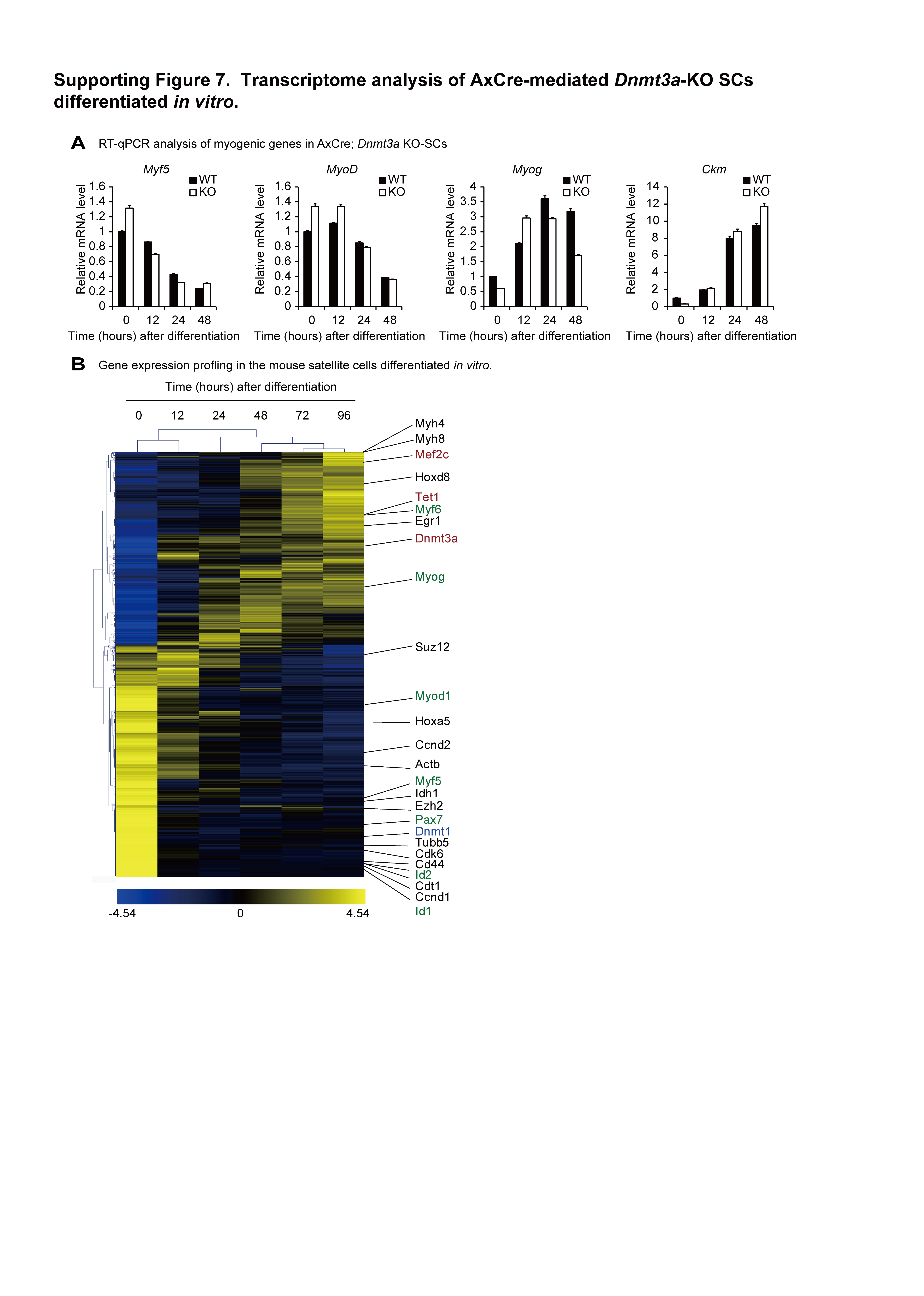

Supplement: S7 Fig — (A) RT-qPCR analysis of myogenic genes in Ax-Cre; Dnmt3a-KO and WT SCs during differentiation. (B) A heat map of the gene expression profile showing the result of microarray analysis of WT SCs differentiated in vitro. (TIF) [file pgen.1006167.s007.tif]

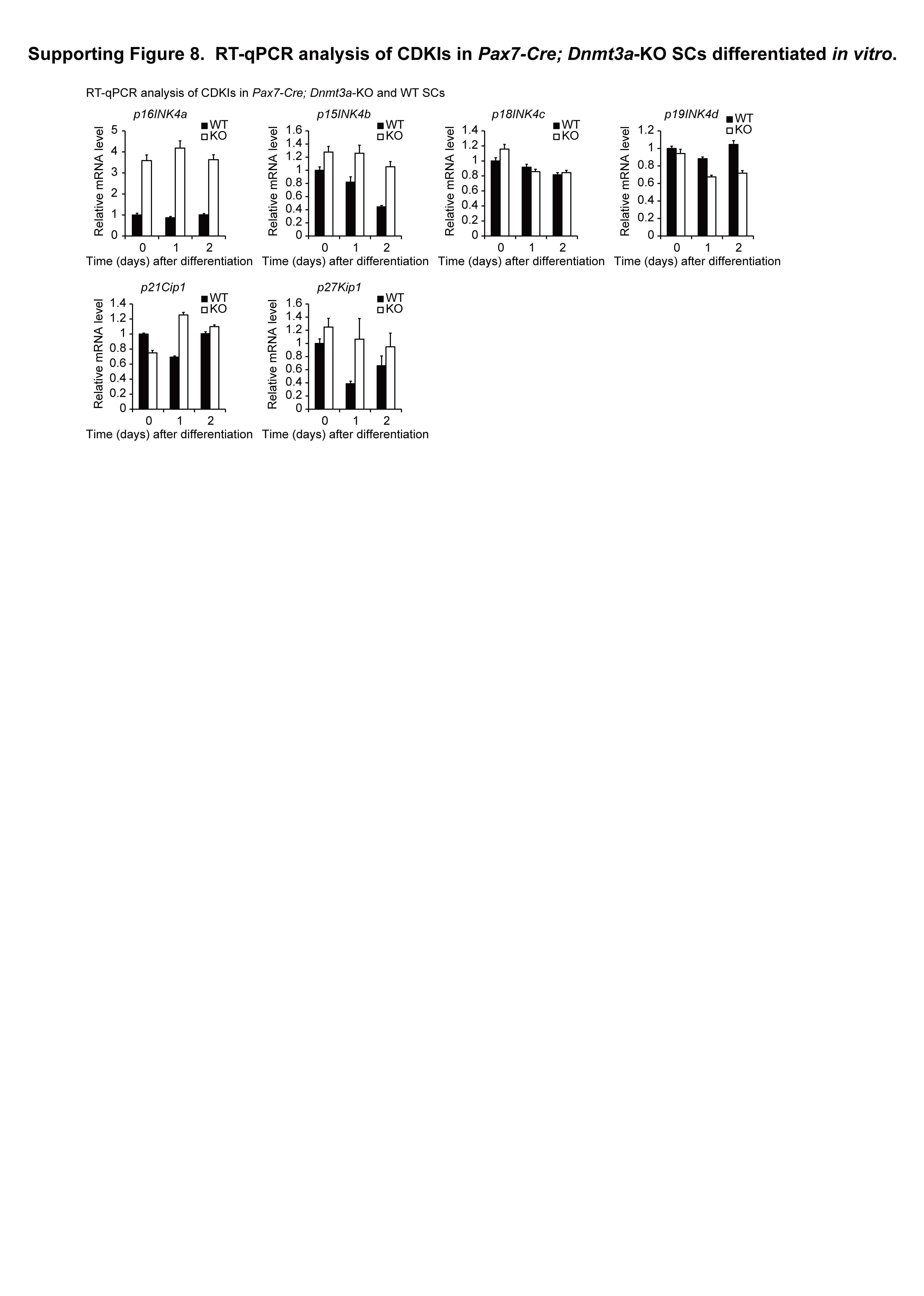

Supplement: S8 Fig — RT-qPCR analysis of CDKIs in Pax7-Cre; Dnmt3a-KO and WT SCs during differentiation. Dnmt3a-KO SCs express p16INK4a at higher level than WT SCs. (TIF) [file pgen.1006167.s008.tif]

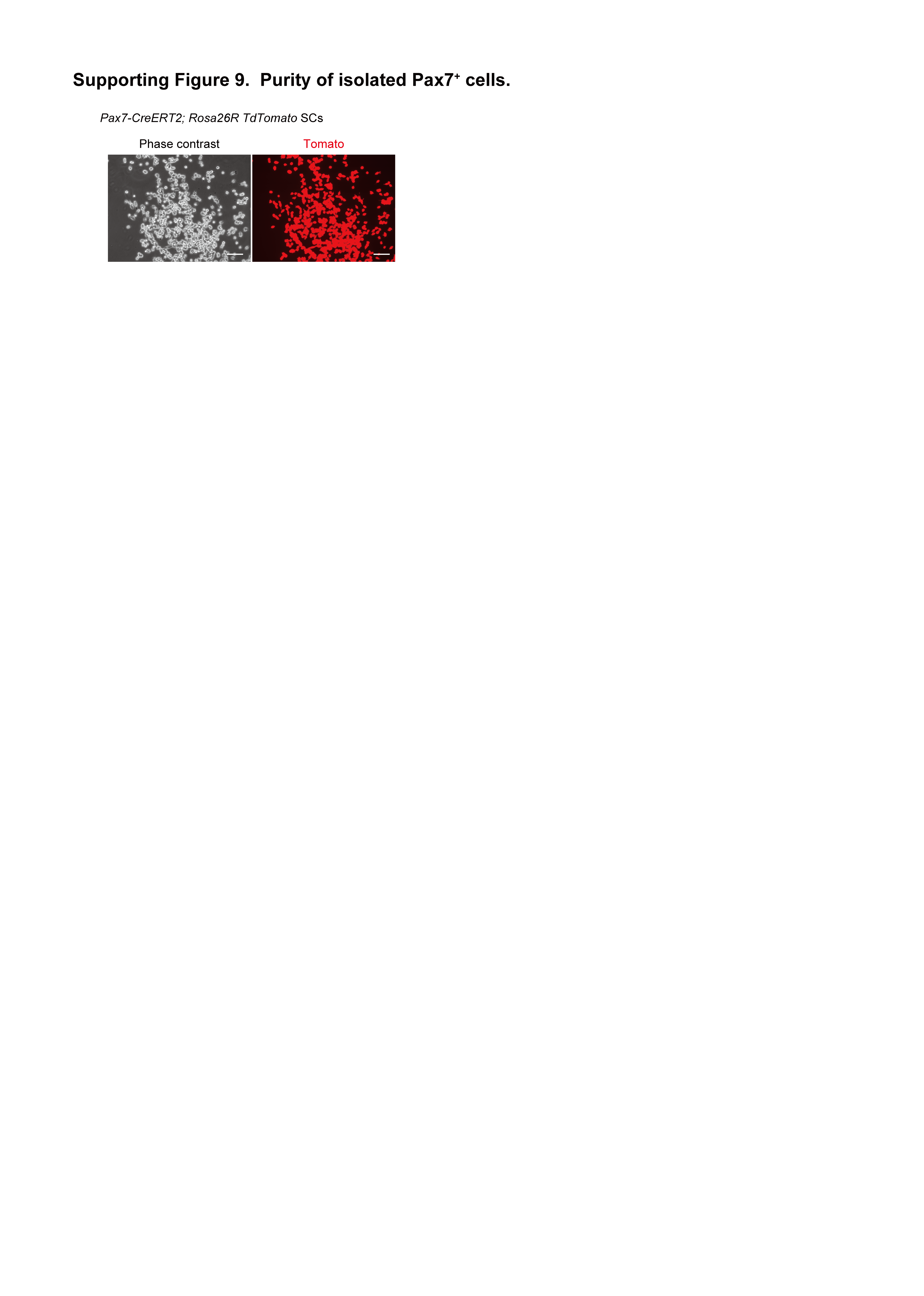

Supplement: S9 Fig — Single myofibers were harvested from Pax7-CreERT2; Rosa26R TdTomato mice after tamoxifen administration. All the cells that have migrated from the myofibers are RFP positive, Scale bar—200 μm. (TIF) [file pgen.1006167.s009.tif]

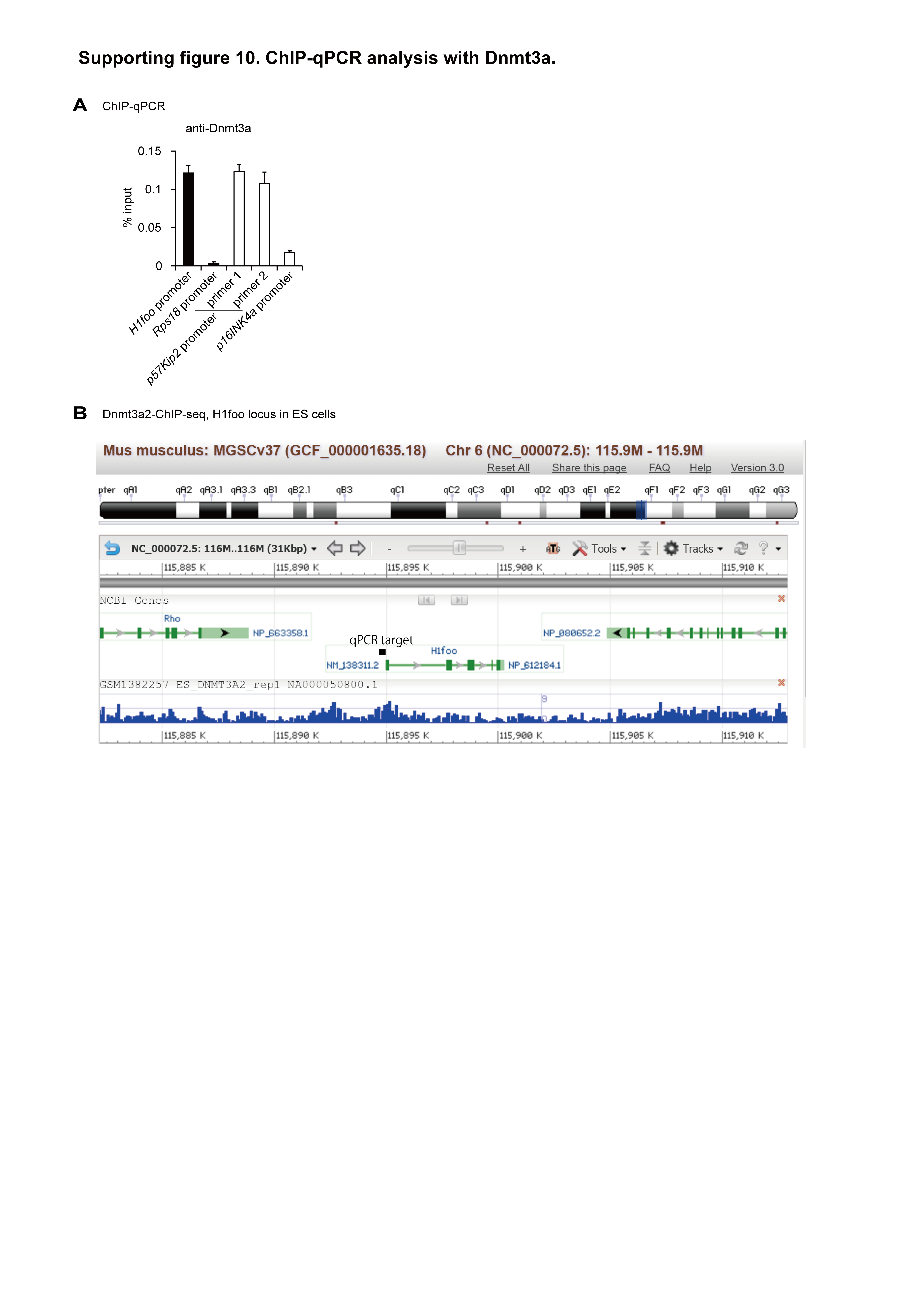

Supplement: S10 Fig — (A) Enrichment of p57Kip2 promoter region is as high as H1foo promoter region. Two different pairs of primers are used for the p57Kip2 promoter region. All primer sequences are listed in S1 Table. (B) Reanalysis of Dnmt3a2-ChIP-seq (GSE57413). Dnmt3a2-ChIP-seq data in ES cells. Results around the H1foo locus is shown. The primers for the ChIP in the H1foo locus were designed on the basis of Dnmt3a2-ChIP-seq data by Baubec et.al [49]. (TIF) [file pgen.1006167.s010.tif]

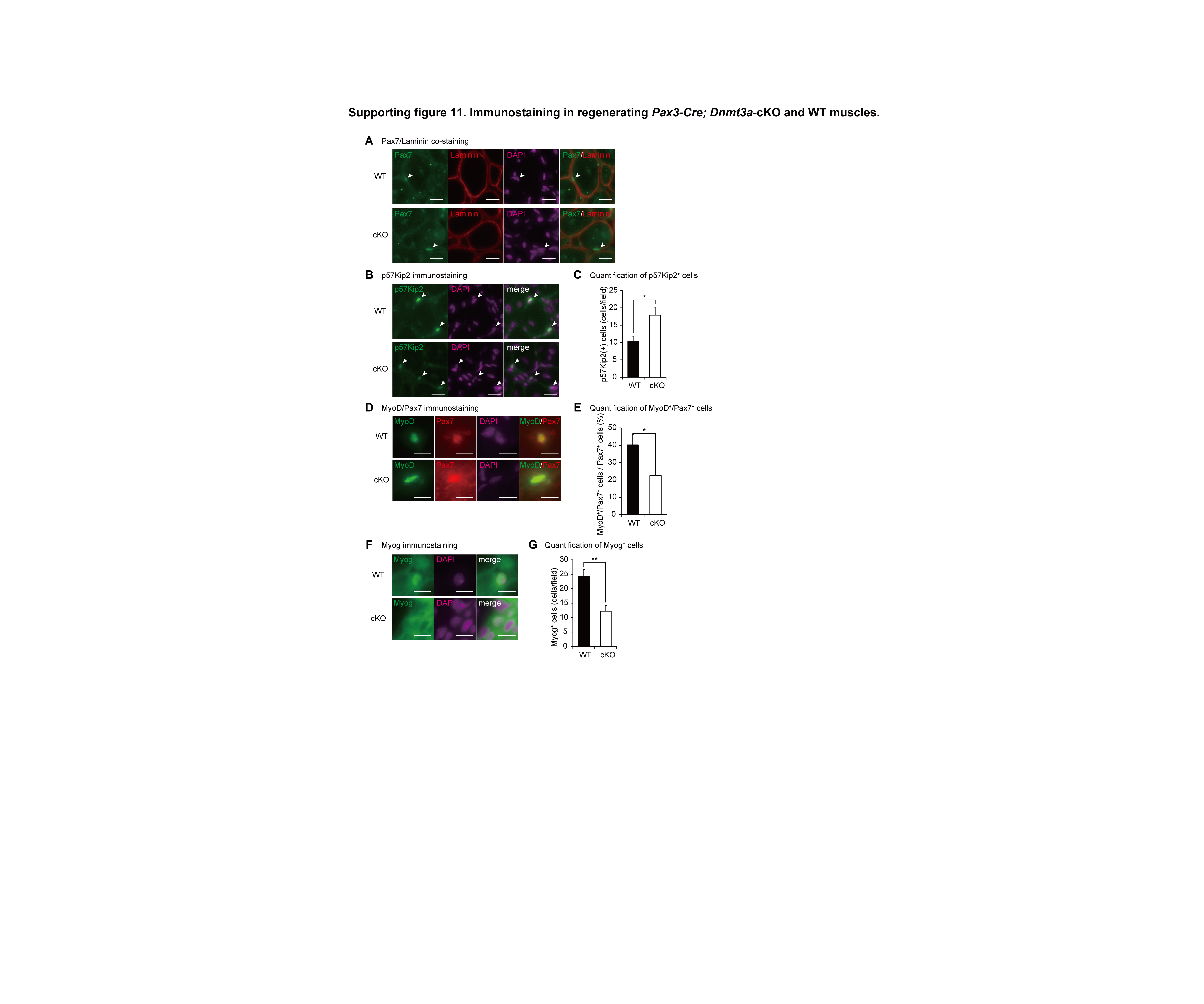

Supplement: S11 Fig — (A) Pax7/Laminin co-staining. Pax7+ cells are located inside the basal lamina in the regenerating myofibers, representing that SCs are stained by an anti-Pax7 antibody. Arrowheads indicate Pax7+ nuclei. Scale bar—20 μm. (B) p57Kip2 immunostaining. Arrowheads indicate p57Kip2+ nuclei. Scale bar—20 μm. (C) Quantification of p57Kip2+ cells; *p<0.05, Student’s t-test. (D) MyoD/Pax7 co-staining. Scale bar—10 μm. (E) Quantification of MyoD+Pax7+ cells. The ratios of MyoD+/Pax7+ cells to a total number of Pax7+ cells in each field of view are shown; *p<0.05, Student’s t-test. (F) Myog immunostaining. Scale bar—10 μm. (G) Quantification of Myog+ cells; **p<0.01, Student’s t-test. (TIF) [file pgen.1006167.s011.tif]

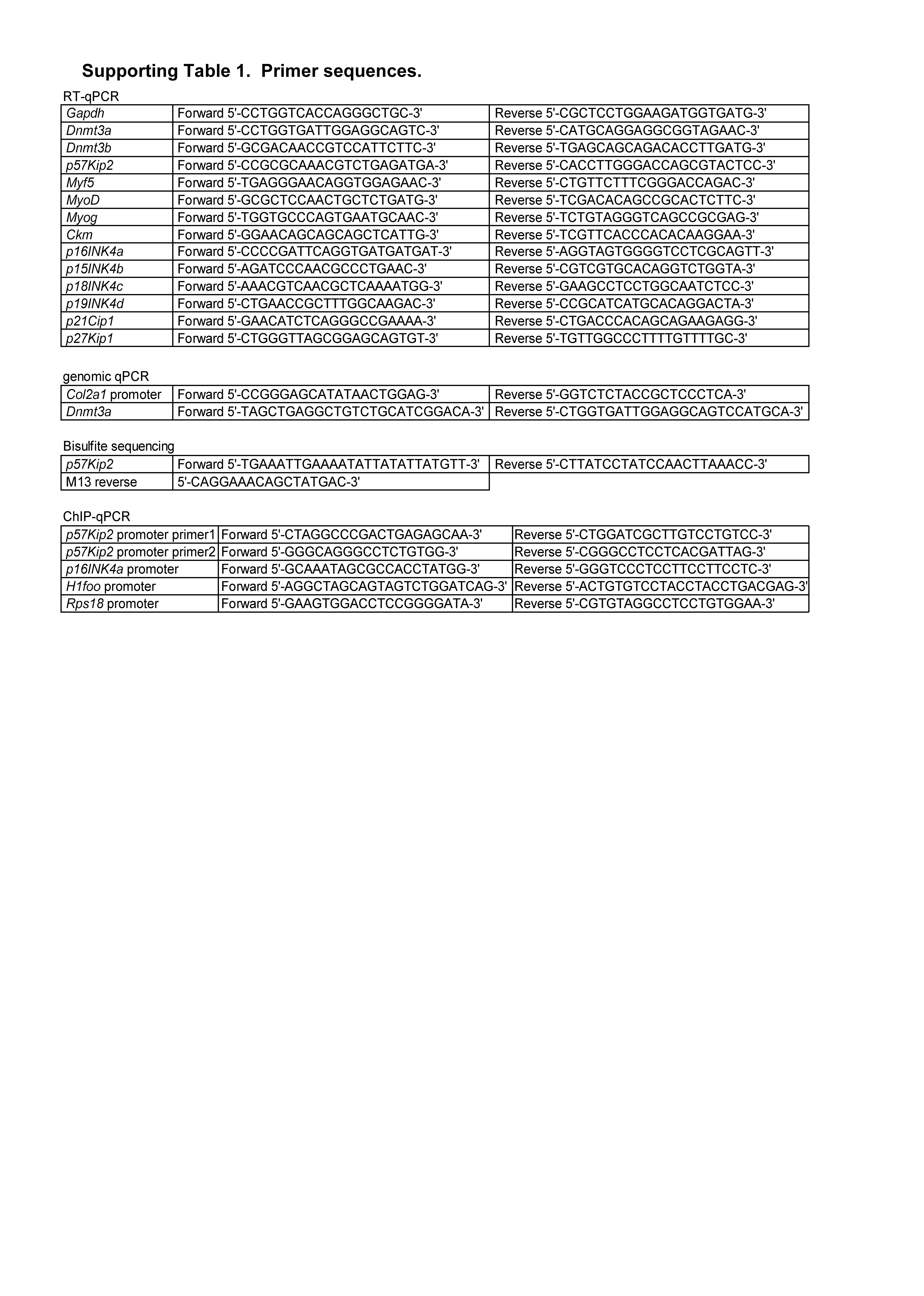

Supplement: S1 Table — (TIF) [file pgen.1006167.s012.tif]
